# Supplementary material for: Structural Thermokinetic Modelling
Source: Metabolites. 2022 May 11;12(5):434. doi: 10.3390/metabo12050434 (PMC9144996; doi:10.3390/metabo12050434)
Supplement: Supplementary file 1 [file metabolites-12-00434-s001.zip › metabolites-1648792-supplementary-english-WL.zip!/metabolites-1648792-supplementary-english-WL.pdf]

Structural Thermokinetic Modelling

Supplementary Text

Wolfram Liebermeister

Université Paris-Saclay, INRAE, MaIAGE, 78350 Jouy-en-Josas, France

Contents

S1 Mathematical Symbols **2**

S2 Kinetic Models and Reaction Elasticities **3**

S2.1 Kinetic Models . . . 3

S2.2 Reaction Elasticities and Thermodynamics . . . 6

S2.3 Metabolic Control Theory . . . 9

S2.4 Model Construction by STM . . . 10

S3 Model Ensembles **13**

S3.1 Sampling the Model Variables . . . 13

S3.2 Do Model Variants Differ in Their Behaviour? Some Useful Statistical Tests . . . 14

S3.3 Extensions of STM . . . 16

S3.4 Example Model: Glycolysis in Human Hepatocytes . . . 18

S4 Metabolic Synergies and Fluctuations **19**

S4.1 Synergies Between Static Perturbations . . . 19

S4.2 Metabolic Fluctuations . . . 21

S4.3 Role of Thermodynamics and Enzyme Saturation in Metabolic Control and Fluctuations . . . 23

S5 Mathematical Derivations **24**

S5.1 Probability Densities for Saturation Values and Dissociation Constants . . . 24

S5.2 Metabolic Control and Response Coefficients . . . 25

S5.3 Spectral Power Density and Temporal Variability Due to Chemical Noise . . . 26

S5.4 Synergy Effects . . . 28

S5.5 Response and Synergy Coefficients . . . 28

S1 Mathematical Symbols

**Network**	
Stoichiometric matrix (all metabolites)	$\mathbf{N}^{\mathrm{tot}}$
Stoichiometric matrix (external metabolites)	$\mathbf{N}^{\mathrm{ext}}$
Stoichiometric matrix (internal metabolites)	$\mathbf{N} = \mathbf{L}\,\mathbf{N}^{\mathrm{ind}}$
Link matrix	$\mathbf{L}$
Stoichiometric matrix (independent internal metabolites)	$\mathbf{N}^{\mathrm{ind}}$
Cooperativity coefficient	h_l
Stoichiometric coefficient	n_{il}
Reactant molecularity	$m_{li}^{\pm} = h_l\,\lvert n_{il}\rvert$
Activation coefficient	m_{li}^{A}
Inhibition coefficient	m_{li}^{I}
**Metabolic Variables**	
Flux	v_l
Internal metabolite concentration	c_i
External metabolite concentration	x_j
Enzyme level	e_l
**Thermodynamic Variables**	
Mass-action ratio	$q_l^{\mathrm{ma}} = \prod_i c_i^{n_{il}}$
Equilibrium constant	$k_l^{\mathrm{eq}} = \prod_i (c_i^{\mathrm{eq}})^{n_{il}}$
Standard chemical potential	μ_i°
Chemical potential	$\mu_i = \mu_i^{\circ} + RT\,\ln c_i$
Thermodynamic force	$\theta_l = -\Delta_{\mathrm{r}}\mu_l/RT$
One-way flux ratio ζ_l	$\zeta_l = v_{+l}/v_{-l} = \mathrm{e}^{h_l\,\theta_l}$
**Rate Laws**	
Rate law	$\nu_l(\mathbf{c},\mathbf{e},\mathbf{x}) = e_l\,k_l(\mathbf{c},\mathbf{x})$
Michaelis–Menten constant	k_{li}^{M}
Activation constant	k_{li}^{A}
Inhibition constant	k_{li}^{I}
Catalytic constant	$k_{\pm,l}^{\mathrm{cat}}$
Maximal velocity	$v_{\pm l}^{\mathrm{max}} = e_l\,k_{\pm,l}^{\mathrm{cat}} = \max_{\mathbf{c},\mathbf{x}}\nu_l(\mathbf{c},\mathbf{e},\mathbf{x})$
Velocity constant	$k_l^{\mathrm{V}} = \sqrt{k_{+l}^{\mathrm{cat}}\,k_{-l}^{\mathrm{cat}}}$
**Elasticity Sampling**	
Saturation value	$\beta_{li}^{\mathrm{M}}, \beta_{li}^{\mathrm{A}}, \beta_{li}^{\mathrm{I}}$
Unscaled elasticity	$E_{c_i}^{v_l} = \frac{\partial \nu_l}{\partial c_i}$
Scaled elasticity	$\hat{E}_{c_i}^{v_l} = \frac{\partial \ln \lvert\nu_l\rvert}{\partial \ln c_i}$
**Metabolic Control Theory**	
Steady-state flux	$v_l = v_l^{\mathrm{st}}(\mathbf{e},\mathbf{x})$
Steady-state concentration	$c_i = c_i^{\mathrm{st}}(\mathbf{e},\mathbf{x})$
Jacobian matrix (independent metabolites)	$\mathbf{A} = \mathbf{N}^{\mathrm{ind}}\,\mathbf{E}_{\mathrm{c}}\,\mathbf{L}$
Unscaled response coefficient	$R_{e_l}^{c_i} = \frac{\partial c_i}{\partial e_l}, R_{e_l}^{v_j} = \frac{\partial v_j}{\partial e_l}$
Unscaled control coefficients	$C_{v_l}^{c_i} = R_{e_l}^{c_i}/E_{e_l}^{v_l}, C_{v_l}^{v_j} = R_{e_l}^{v_j}/E_{e_l}^{v_l}$
Scaled response/control coefficient	$\hat{R}_{e_l}^{c_i} = \hat{C}_{v_l}^{c_i} = \frac{\partial \ln c_i}{\partial \ln e_l}, \hat{R}_{e_l}^{v_j} = \hat{C}_{v_l}^{v_j} = \frac{\partial \ln \lvert v_j\rvert}{\partial \ln e_l}$

Table S1: Symbols used in STM. Network elements are denoted by i (metabolites) and l (reactions). Second-order elasticities, response coefficients (called synergy coefficients), and control coefficients are defined similarly to first-order coefficients.

S2 Kinetic Models and Reaction Elasticities

S2.1 Kinetic Models

Metabolic networks and kinetic models A metabolic network is defined by a set of chemical reactions and regulatory arrows pointing from metabolites to reactions (Figure S2.1 (a)). The molecularities[1] $m^{\rm S}_{li}$ (for substrates) and $m^{\rm P}_{li}$ (for products) are given by the stoichiometric coefficients n_{li} between metabolite i and enzyme l, multiplied by the reaction's cooperativity coefficient h_l (i.e., $h_l\, n_{li} = m^{\rm S}_{li} - m^{\rm P}_{li}$) [1]. The stoichiometric coefficients and regulation coefficients $m^{\rm A}_{li}$ and $m^{\rm I}_{li}$ (activation: $m^{\rm A}_{li} = 1$; inhibition: $m^{\rm I}_{li} = 1$; zero values otherwise) are collected in matrices that define the network. Kinetic models [2] describe reaction rates by rate laws $\nu_l(\mathbf{c}, \mathbf{e}, \mathbf{x}) = e_l\, k_l(\mathbf{c}, \mathbf{x})$ (Figure S2.1 (b)). Modular rate laws [1] (see below) contain two types of kinetic constants: catalytic constants $k^{\rm cat}_{\pm}$ (in s^{-1}) describe the speed of the forward and backward rates, while metabolite-enzyme binding is described by dissociation constants $k^{\rm M}$ for reactants, activation constants $k^{\rm A}$ for activators, and inhibition constants $k^{\rm I}$ for inhibitors (in mM). For each reaction, a certain ratio of all these constants must be equal to the equilibrium constant (Haldane relationship). Given stoichiometric matrix and rate laws, we obtain the dynamic rate equations ${\rm d}c_i/{\rm d}t = \frac{1}{V_i} \sum_l n_{il}\, \nu_l(\mathbf{c}, \mathbf{e}, \mathbf{x})$ for internal metabolite concentrations c_i, with external metabolite concentrations x_j and enzyme levels e_l as parameters. We assume that all metabolites i are homogeneously distributed within cell compartments of constant size V_i. Metabolite concentrations are given in mM = mol/m^3, reaction rates as amounts per time (mol/s), enzyme levels as amounts (mol), and volumes in m^3. In single-compartment models, we may choose a compartment size of 1 (dimensionless) and measure reaction rates in mM/s and enzyme levels in mM. If we analyse steady states, the choice of flux units does not play a role.

Modular rate laws Modular rate laws [1] are generic reversible rate laws that capture various reaction stoichiometries, enzyme mechanisms, and types of regulation by effector molecules. Formulae for different rate laws and their elasticities can be found in the Supplementary Materials of [1]. As an example, let us consider a reaction $A + B \leftrightharpoons 2C$ without effectors. The common modular (CM) rate law is a reversible Michaelis–Menten kinetics, generalised for arbitrary stoichiometries. With two substrates A and B and one product C, it reads

$$\nu(a,b,c,e) = e\, \frac{k^{\rm cat}_{+}\,(a/k^{\rm M}_{\rm A}), (b/k^{\rm M}_{\rm B}) - k^{\rm cat}_{-}\,(c/k^{\rm M}_{\rm C})^2}{(1 + a/k^{\rm M}_{\rm A})(1 + b/k^{\rm M}_{\rm B}) + (1 + c/k^{\rm M}_{\rm C})^2 - 1} \tag{S1}$$

with reactant constants $k^{\rm M}_{\rm A}$, $k^{\rm M}_{\rm B}$, and $k^{\rm M}_{\rm C}$ (in mM) and catalytic constants $k^{\rm cat}_{+}$ and $k^{\rm cat}_{-}$ (in s^{-1}) for forward and backward direction. Modular rate laws can be adapted to different types of reactions and enzymes: if an enzyme is regulated by an effector molecule, this can be described, for example, by prefactors $\beta^{\rm A}_{\rm X} = \frac{x/k^{\rm A}_{\rm X}}{1+x/k^{\rm A}_{\rm X}}$ for activators X or $\alpha^{\rm I}_{\rm Y} = \frac{1}{1+y/k^{\rm I}_{\rm Y}}$ for inhibitors Y. Moreover, with a Hill-like exponent h the rate laws can capture sigmoidal kinetics. Other types of rate laws use the same parameters, but another denominator, for example, the saturable modular (SM) rate law

$$\nu(a,b,c,e) = e\, \frac{k^{\rm cat}_{+}\,(a/k^{\rm M}_{\rm A})\,(b/k^{\rm M}_{\rm B}) - k^{\rm cat}_{-}\,(c/k^{\rm M}_{\rm C})^2}{(1 + a/k^{\rm M}_{\rm A})(1 + b/k^{\rm M}_{\rm B})(1 + c/k^{\rm M}_{\rm C})^2}. \tag{S2}$$

In the denominator, the substrate and product terms are simply multiplied. Modular rate laws assume an enzyme mechanism in which substrates and products bind rapidly, independently, and in random order. The

[1] The molecularities resemble stoichiometric coefficients, but with a slight difference: while stoichiometric cofficients (in a reaction sum formula) can be rescaled, the molecularities are actual molecule numbers in the enzyme mechanism, and therefore uniquely determined. They can be written as the (absolute) stoichiometric cofficients multiplied by a cooperativity exponent h_l for each reaction (typically $h_l = 1$).) [1]. In the formula for thermodynamic forces, $\boldsymbol{\theta} = {\mathbf{N}^{\rm tot}}^{\top} \boldsymbol{\mu}/RT$, we tacitly assume that stoichiometric coefficients are given by molecularities. Otherwise, this formula must contain h_l as a prefactor.

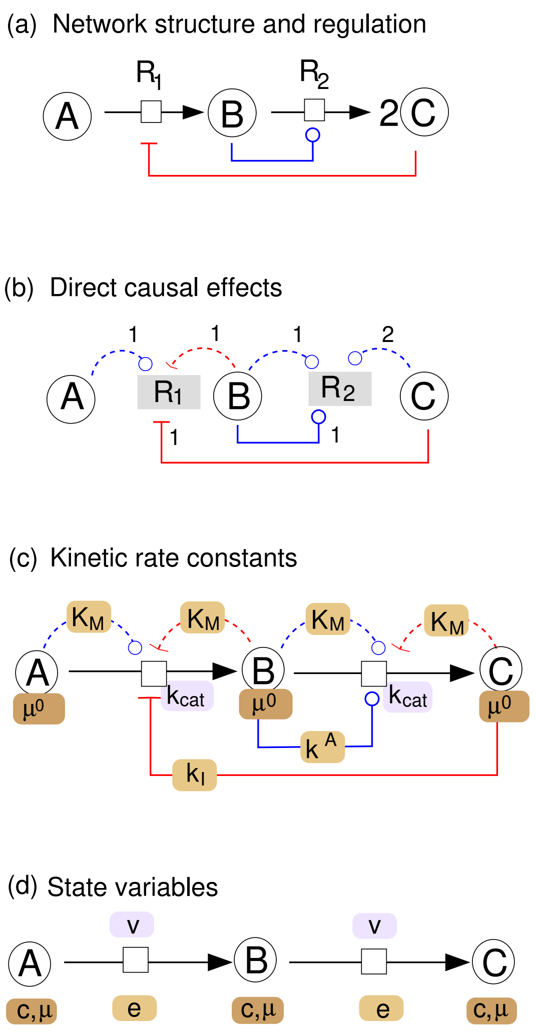

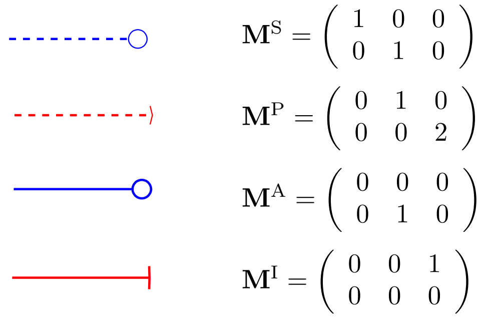

Figure S1: Structure and kinetic description of metabolic networks. (a) Metabolic network and structure matrices. Our metabolic pathway consists of two reactions $A \leftrightharpoons B$ and $B \leftrightharpoons 2\,C$. Reaction R_1 is inhibited by the end product C, while reaction R_2 is activated by its own substrate B; (b) pathway shown as a bipartite network. Each arrow carries a number: dotted arrows show stoichiometric coefficients for substrates and products, regulation arrows (solid) carry coefficients of 1. Right: the arrows and coefficients can be represented by network matrices $\mathbf{M}^{\mathrm{S}}$, $\mathbf{M}^{\mathrm{P}}$, $\mathbf{M}^{\mathrm{A}}$, and $\mathbf{M}^{\mathrm{I}}$ whose rows and columns correspond to reactions and metabolites, respectively. $\mathbf{M}^{\mathrm{S}}$ and $\mathbf{M}^{\mathrm{P}}$ follow directly from the stoichiometric matrix; (c) kinetic constants in modular rate laws. The standard chemical potentials μ_i° of metabolites determine the equilibrium constants. All stoichiometry or regulation arrows are associated with dissociation constants k_{li}^{M}, k_{li}^{A}, or k_{li}^{I}. The velocity constants $k_l^{\mathrm{V}} = \sqrt{k_{+l}^{\mathrm{cat}}\,k_{-l}^{\mathrm{cat}}}$ are assigned to reactions. Together, the constants determine the catalytic constants $k_{\pm,l}^{\mathrm{cat}}$. (d) A metabolic state is characterised by fluxes v_l, metabolite concentrations c_i, chemical potentials $\mu_i = \mu_i^\circ + RT\,\ln c_i$, and enzyme levels e_l.

reactant constants k_{li}^{M} are dissociation constants of the elementary binding steps. Like the k^{M} values in Michaelis–Menten kinetics, they denote reactant concentrations that would yield a half-maximal saturation (or $1/|n_{il}|$-maximal saturation if $|n_{il}| > 1$). The catalytic constants $k_{\pm}^{\mathrm{cat}}$ stem from the slow conversion step between substrate and product molecules. In all modular rate laws, the kinetic constants are required to satisfy Haldane relationships. One way to ensure this is to predefine the equilibrium constants, to treat the velocity constants k^{V} as free parameters, and to compute the catalytic constants by using Equation (S9).

Thermodynamic laws The kinetics and steady states of metabolic systems are constrained by thermodynamic, which defines relationships between metabolite concentrations c_i, reaction rates v_l, equilibrium constants k_l^{eq}, and chemical potentials μ_i. The chemical potentials μ_i are defined as the derivatives $\mu_i = \partial G/\partial n_i$ of the system's total Gibbs free energy by the metabolite mole numbers n_i. For ideal mixtures (metabolites at low concentrations in an aqueous solution, no mixture effects, activity coefficients of 1), the chemical potential of metabolite i is given by the formula

$$\mu_i \quad = \quad \mu_i^\circ + RT\,\ln c_i/c_{\mathrm{std}} \qquad \text{(S3)}$$

with Boltzmann gas constant R, absolute temperature T, and chemical potential μ_i° of metabolite i at standard concentration[2] $c_{\rm std}$. Here I omit the division by $c_{\rm std}$, assuming that all concentrations are given in units of the standard concentration. The ratio $q_l^{\rm ma} = \prod_i (c_i)^{n_{il}}$ of product and substrate concentrations for a reaction is called mass–action ratio. In chemical equilibrium states, this ratio always has the same value, called equilibrium constant $k_l^{\rm eq}$, which can be written as $k^{\rm eq} = {\rm e}^{-\Delta\mu^\circ/RT}$. The thermodynamic force θ_r, in a (possibly non-steady) metabolic state is defined as

$$\theta_l \quad = \quad -\frac{1}{RT}\Delta_{\rm r}\mu_l = -\frac{1}{RT}\sum_i \mu_i\, n_{il}. \tag{S4}$$

Note that, in this definition, the prefactors in the difference Δ refer to actual molecularities, not to arbitrarily scaled stoichiometric coefficients. A driving force θ describes the Gibbs free energy dissipation (in kJ/mol) associated with a reaction event and can be computed from the equilibrium constant $k_l^{\rm eq}$ and the mass–action ratio $q_l^{\rm ma}$ (for reaction l):

$$\theta_l = -\frac{1}{RT}\sum_i n_{il}\,\mu_i = \ln k_l^{\rm eq}/q_l^{\rm ma}. \tag{S5}$$

In generalised Michaelis–Menten rate laws, the ratio of forward and backward rates $v_l^\pm$ of reaction l is given by

$$\zeta_l = v_{+l}/v_{-l} = {\rm e}^{h_l\,\theta_l} = k^{\rm eq}/\prod c_i^{n_i}. \tag{S6}$$

The thermodynamic-kinetic formalism [3] defines the thermokinetic potential $\xi_i = {\rm e}^{\mu_i/RT}$ and splits it into $\xi_i = C_i\,c_i$, where C_i is called capacity. For ideal mixtures (satisfying Equation (S3)), the thermokinetic potential is given by $\xi_i = {\rm e}^{\mu_i^\circ/RT}\,c_i$ with a capacity $C_i = {\rm e}^{\mu_i^\circ/RT}$, and related to ζ_l via

$$\zeta_l = {\rm e}^{h_h\,\theta_l} = {\rm e}^{-h_h\,\Delta_{\rm r}\mu_l} = \prod_i ({\rm e}^{\mu_i/RT})^{-h_l\,n_{il}} = \prod_i \xi_i^{-h_l\,n_{il}}. \tag{S7}$$

In kinetic models, thermodynamic laws impose three sorts of constraints: a relation between flux directions and thermodynamic forces; Wegscheider conditions for equilibrium constants; and Haldane relationships between equilibrium constants and kinetic parameters. These constraints arise as follows: (i) To carry a non-zero flux, chemical reactions must show a positive production of entropy per volume and time, $\sigma_l = v_l\,A_l/T = Rv_l\,\theta_l$ (with the reaction affinity $A = -\Delta_{\rm r}G$). As a consequence, a (non-zero) reaction rate v_l and the corresponding thermodynamic force θ_l must point in the same direction. (ii) The vector $\ln \mathbf{k}^{\rm eq}$ of logarithmic equilibrium constants can be written as $\ln \mathbf{k}^{\rm eq} = {\mathbf{N}^{\rm tot}}^\top \ln \mathbf{c}^{\rm eq}$, with a vector $\mathbf{c}^{\rm eq}$ of metabolite concentrations in an equilibrium state. For any such vector $\ln \mathbf{k}^{\rm eq}$, the Wegscheider conditions $\mathbf{K}^\top \ln \mathbf{k}^{\rm eq} = 0$ have to be satisfied [4, 5], where $\mathbf{K}$ is a null space matrix satisfying $\mathbf{N}\,\mathbf{K} = 0$. Similar Wegscheider conditions hold for all vectors of the form $\mathbf{x} = \mathbf{N}^{\rm tot}\,\mathbf{y}$, including logarithmic mass–action ratios $\ln q_l^{\rm ma}$ and thermodynamic forces θ_l. (iii) The fact that reaction rates $v_l(\mathbf{c}^{\rm eq}, \mathbf{e})$ vanish at chemical equilibrium implies a Haldane relationship between equilibrium constant and kinetic constants [6, 3]. For all modular rate laws, the Haldane relationship reads

$$k_l^{\rm eq} \quad = \quad k_{+l}^{\rm cat}/k_{-l}^{\rm cat}\prod_i (k_{li}^{\rm M})^{n_{il}}. \tag{S8}$$

[2] For kinetic models, it is convenient to use a standard concentration of 1 mM, equal to the measurement unit for concentrations. In thermodynamic flux analysis, the common standard concentration is 1M. The conversion between the two conventions requires an adjustment of reaction Gibbs free energies, standard Gibbs free energies of formation, and equilibrium constants.

To construct parameter sets that satisfy this relation, we define the turnover constants $k_l^{\mathrm{V}} = \sqrt{k_{+l}^{\mathrm{cat}}\, k_{-l}^{\mathrm{cat}}}$ as the geometric means of forward and backward catalytic constants. By rewriting Equation (S8), we can now express the forward and backward catalytic constants as

$$k_{\pm,l}^{\mathrm{cat}} \;=\; k_l^{\mathrm{V}}\,(k_l^{\mathrm{eq}} \prod_i (k_{li}^{\mathrm{M}})^{-n_{il}})^{\pm 1/2}. \tag{S9}$$

These values satisfy the Haldane relationship by construction.

S2.2 Reaction Elasticities and Thermodynamics

Scaled and unscaled reaction elasticities The derivatives between kinetic laws $\nu_l(\cdot)$ and enzyme concentrations e_p, metabolite concentrations c_j, or other function arguments are called reaction elasticities (see Figure 1 (a) in main text). Given a rate law $\nu_l(e_l, \mathbf{c})$, the unscaled reaction elasticities are defined by derivatives

$$E_{c_i}^{v_l} = \frac{\partial \nu_l}{\partial c_i}, \quad E_{c_i c_j}^{v_l} = \frac{\partial^2 \nu_l}{\partial c_i\, \partial c_j}, \tag{S10}$$

while the corresponding scaled elasticities are defined by logarithmic derivatives

$$\hat{E}_{c_i}^{v_l} = \frac{\partial \ln |\nu_l|}{\partial \ln c_i}, \quad \hat{E}_{c_i c_j}^{v_l} = \frac{\partial^2 \ln |\nu_l|}{\partial \ln c_i\, \partial \ln c_j}. \tag{S11}$$

Elasticities for other arguments of the rate law function (e.g., the enzyme level e_l instead of c_i) are defined accordingly. Scaled elasticities are dimensionless and can be seen as effective reaction orders: for mass–action kinetics, they are given by the substrate molecularities; for an enzyme that is fully saturated with the metabolite in question, they vanish. Scaled and unscaled elasticities can be interconverted by

$$\hat{E}_{c_i}^{v_l} = \frac{c_i}{v_l} E_{c_i}^{v_l}, \qquad \hat{E}_{c_i c_j}^{v_l} = \frac{c_i\, c_j}{v_l} E_{c_i c_j}^{v_l} - \frac{c_i\, c_j}{v_l^2} E_{c_i}^{v_l} E_{c_j}^{v_l} + \delta_{ij} \frac{c_i}{v_l} E_{c_i}^{v_l} \tag{S12}$$

and

$$E_{c_i}^{v_l} = \frac{v_l}{c_i}\, \hat{E}_{c_i}^{v_l}, \qquad E_{c_i c_j}^{v_l} = \frac{v_l}{c_i\, c_j} \left[\hat{E}_{c_i c_j}^{v_l} + \hat{E}_{c_i}^{v_l}\, \hat{E}_{c_j}^{v_l} - \delta_{ij} \hat{E}_{c_i}^{v_l} \right]. \tag{S13}$$

Analogous conversion formulae hold for all types of sensitivities, including elasticities with respect to other parameters, control coefficients, and response coefficients [7, 8].

Elasticities and thermodynamic force Elasticities depend on the rate laws, but also on thermodynamics. In reversible rate laws, the net reaction rate $v_l = v_{+l} - v_{-l}$ is the difference of forward and backward rates, whose ratio $v_{+l}/v_{-l} = \mathrm{e}^{\theta_l}$ is determined by the thermodynamic force θ_l. The thermodynamic force, in turn, depends on reactant concentrations and equilibrium constant as $\theta_l = -\Delta_{\mathrm{r}} G_l / RT = \ln \frac{k^{\mathrm{eq}}}{\prod_i c_i^{n_{il}}}$ (see Figure 1 (b) in main text). If the thermodynamic force is large, the forward flux dominates and the net rate becomes sensitive to substrate fluctuations, but less sensitive to product fluctuations; therefore, the substrate elasticity increases and the product elasticity decreases. Near chemical equilibrium, where thermodynamic forces come close to zero, the scaled elasticities go to infinity.

Elasticities of modular rate laws The scaled elasticities of the SM rate law Equation (S2) contain the thermodynamic term as well as four terms that correspond to substrates, products, activators, and inhibitors:

$$\hat{E}^{v_l}_{c_i} = \frac{\zeta_l\, m^{\rm S}_{li} - m^{\rm P}_{li}}{\zeta_l - 1} - m^{\rm S}_{li}\alpha^{\rm M}_{li} - m^{\rm P}_{li}\beta^{\rm M}_{li} + m^{\rm A}_{li}\,\alpha^{\rm A}_{li} - m^{\rm I}_{li}\,\beta^{\rm I}_{li}. \tag{S14}$$

For near-equilibrium reactions (small θ_l) and for strongly driven reactions ($|\theta_l| \rightarrow \infty$), the first three terms can be approximated by

$$\begin{aligned} |\theta_l| \approx 0 &\;:\; \frac{1}{\theta_l}(m^{\rm S}_{li} - m^{\rm P}_{li}) + m^{\rm S}_{li}\alpha^{\rm M}_{li} - m^{\rm P}_{li}\beta^{\rm M}_{li} \\ \theta_l \rightarrow \infty &\;:\; (m^{\rm S}_{li} - m^{\rm P}_{li})\,{\rm e}^{-\theta_l} + m^{\rm S}_{li}\alpha^{\rm M}_{li} - m^{\rm P}_{li}\beta^{\rm M}_{li} \\ \theta_l \rightarrow -\infty &\;:\; (m^{\rm P}_{li} - m^{\rm S}_{li}){\rm e}^{-|\theta_l|} - m^{\rm S}_{li}\,\beta^{\rm M}_{li} + m^{\rm P}_{li}\alpha^{\rm M}_{li}. \end{aligned} \tag{S15}$$

The last two terms in these formulae represent exactly the formula used in SKM [9], while the first term employs a thermodynamic correction. The scaled elasticities of the common modular rate law are a bit more complicated:

$$\hat{E}^{v_l}_{c_j} = \beta_{lj}\frac{\zeta_l\, m^{\rm S}_{lj} - m^{\rm P}_{lj}}{\zeta_l - 1} - \beta_{lj}\frac{m^{\rm S}_{lj}\psi^{+}_{l} + m^{\rm P}_{lj}\psi^{-}_{l}}{\psi^{+}_{l} + \psi^{-}_{l} - 1} + m^{\rm A}_{li}\,\alpha^{\rm A}_{li} - m^{\rm I}_{li}\,\beta^{\rm I}_{li}, \tag{S16}$$

where $\psi^{\pm}_{l} = \prod_l (1{+}c_i/k^{\rm M}_{li})^{m^{\pm}_{li}}$ (see [1]). Formulae for second-order elasticities, unscaled elasticities, parameter elasticities, and other types of modular rate laws can be found in [1].

Elasticities of factorised rate laws (derivation of Equation (8)) For the factorized rate laws, and assuming a positive flux $v > 0$ for simplicity, we obtain

$$\hat{E}^{v_l}_{c_j} = \frac{\partial \ln v_l}{\partial \ln c_i} = \frac{\partial \ln(1 - {\rm e}^{-\theta_l})}{\partial \ln c_i} + \frac{\partial \ln \eta^{\rm kin}}{\partial \ln c_i}, \tag{S17}$$

where

$$\frac{\partial \ln(1 - {\rm e}^{-\theta_l})}{\partial \ln c_i} = \frac{1}{1 - {\rm e}^{-\theta_l}}(-{\rm e}^{-\theta_l})(-)\partial\theta_l/\partial \ln c_i = \frac{{\rm e}^{-\theta_l}}{1 - {\rm e}^{-\theta_l}}(-n_{il}) = \frac{-1}{{\rm e}^{-\theta_l} - 1}n_{il}. \tag{S18}$$

Any set of saturation values yields a consistent model STM relies on two facts about model parameterisation: first, given consistent fluxes and thermodynamic forces, any choice of the saturation values yields a consistent kinetic model; and second, any consistent model can be constructed like this. This can be proven as follows: consider a kinetic model with modular rate laws and a thermodynamically consistent flux distribution $\mathbf{v}$. For simplicity, enzyme levels are subsumed in the catalytic constants $k^{\rm cat}_{\pm,l}$. As shown in [1], a consistent set of parameters, realising $\mathbf{v}$, can be obtained by the following procedure:

1. Freely choose standard chemical potentials μ°_{i} and determine the equilibrium constants.

2. Determine concentrations c_i such that the signs of the thermodynamic forces agree with the flux directions. If the metabolite concentrations are bounded, this may not always be possible, even if the flux distribution is loopless.

3. Freely choose Michaelis constants $k^{\rm M}_{li}$ and activation and inhibition constants $k^{\rm A}_{li}$ and $k^{\rm I}_{li}$. Given the previously chosen metabolite concentrations, this is equivalent to choosing the saturation constants in the range between 0 and 1 and computing the $k^{\rm X}_{li}$ values from them.

4. Choose preliminary values for the velocity constants $k^{\rm V}_{l}{}'$. Compute the catalytic constants $k^{\rm cat}_{\pm,l}{}'$ from

the Haldane relationships (S8). Use the rate laws to compute the reaction rates v'_l. By construction (due to the thermodynamically feasible metabolite concentrations and thermodynamically consistent rate laws), these rates have the same signs as the predefined fluxes. To match reaction rates and fluxes exactly, we just need to adjust the velocity constants, setting $k^{\rm V}_l = (v_l/v'_l)\, {k^{\rm V}}'_l$.

5. If our flux distribution contains inactive reactions, we can decide, for each of them, whether we assume a vanishing thermodynamic force, a vanishing velocity constant, or a vanishing enzyme level. In the first case, we need to apply the strict energetic feasibility criterion for this reaction (i.e., require that the thermodynamic force vanish); in the other cases, there is no feasibility criterion for the reaction, and the $k^{\rm V}$ value or enzyme level is set to zero.

If this procedure yields correct models, then also all models constructed by STM are correct. In STM, we first determine consistent fluxes v_l and thermodynamic forces θ_l that can be realised by a choice of standard chemical potentials μ_i° and concentrations c_i. Thus, when choosing the saturation constants, any choice is equivalent to a choice of $k^{\rm M}_{li}$, $k^{\rm A}_{li}$, and kI_{li} in the algorithm above; the quantities chosen until this point correspond exactly to the results of step 3; steps 4 and 5 will yield a unique, consistent set of parameters. Therefore, models obtained by STM satisfy all relevant constraints.

Independently sampled elasticities would yield inconsistent models A main problem with SKM is that elasticities are replaced by independent random numbers, which means that the resulting kinetic models may violate constraints. If the forward and backward one-way rates of reactions were independent, and not constrained by thermodynamics, independent sampling would be justified: the elasticities could be directly translated into kinetic constants, and each sampled elasticity matrix would define a specific kinetic model. However, in models with reversible rate laws, independently sampled elasticities lead to inconsistent results. For example, consider a reaction A $\leftrightharpoons$ B with reversible mass–action kinetics $v = k^+\, a - k^-\, b$: the scaled reaction elasticities read $\hat{E}_{\rm A} = k^+\, a/v$ (for substrate A) and $\hat{E}_{\rm B} = k^+\, b/v$ (for product B), so their difference $\hat{E}_{\rm A} - \hat{E}_{\rm B} = 1$ is fixed. If we sample these elasticities independently, this relationship is violated and our sampled values cannot be realised by reversible mass–action rate laws. Similar constraints hold for all thermodynamically consistent reversible rate laws.

There can also be inconsistencies between the elasticities of different reactions. Here is a simple example: the reaction A $\rightarrow$ B is catalysed by two isoenzymes with reversible mass–action kinetics:

$$\begin{aligned} v_1 &= \nu_1(a,b) = k_1^+\, a - k_1^-\, b \\ v_2 &= \nu_2(a,b) = k_2^+\, a - k_2^-\, b. \end{aligned} \tag{S19}$$

The symbols a and b denote the concentrations of A and B, and $k_1^{\pm}$ and $k_2^{\pm}$ denote the rate constants. In each reaction, forward and backward kinetic constants must have the same ratio given by the equilibrium constant:

$$k^{\rm eq} = \frac{k_1^+}{k_1^-} = \frac{k_2^+}{k_2^-}. \tag{S20}$$

Therefore, the scaled elasticity matrix can be written as

$$\hat{\mathbf{E}} = \begin{pmatrix} \frac{\partial \ln \nu_1}{\partial \ln a} & \frac{\partial \ln \nu_1}{\partial \ln b} \\ \frac{\partial \ln \nu_2}{\partial \ln a} & \frac{\partial \ln \nu_2}{\partial \ln b} \end{pmatrix} = \begin{pmatrix} k_1^+\, \frac{a}{v_1} & -k_1^-\, \frac{b}{v_1} \\ k_2^+\, \frac{a}{v_2} & -k_2^-\, \frac{b}{v_2} \end{pmatrix} = \begin{pmatrix} \frac{k^{\rm eq}\, a}{k^{\rm eq}\, a - b} & \frac{-b}{k^{\rm eq}\, a - b} \\ \frac{k^{\rm eq}\, a}{k^{\rm eq}\, a - b} & \frac{-b}{k^{\rm eq}\, a - b} \end{pmatrix} = \begin{pmatrix} \frac{\zeta}{\zeta - 1} & \frac{-1}{\zeta - 1} \\ \frac{\zeta}{\zeta - 1} & \frac{-1}{\zeta - 1} \end{pmatrix} \tag{S21}$$

with $\zeta = k^{\rm eq}/(b/a)$. All four elasticities are determined by the same parameter ζ, so sampling them independently leads to a contradiction.

Dependencies between first- and second-order elasticities For a given modular rate law, the scaled elasticities can be computed from stoichiometric coefficients, thermodynamic forces, and saturation values. The fact that one model detail (e.g., the thermodynamic force of a reaction) can influence different reaction elasticities leads to dependencies between these elasticities: if this model detail is varied, the resulting elasticities will be statistically dependent. Here is an example. When a thermodynamic force becomes larger, the substrate elasticities tend to increase and the product elasticities tend to decrease. When comparing all elasticities in a network, or when comparing different instances of an ensemble model, the relationship between thermodynamic force and elasticities leads to positive correlations among substrate elasticities, positive correlations among product elasticities, and negative correlations between substrate and product elasticities within reactions. Just like first-order elasticities, the second-order elasticities are also dependent. Second-order elasticities $\hat{E}^{v_l}_{c_i c_j}$ tend to be negatively correlated with the product $\hat{E}^{v_l}_{c_i}\,\hat{E}^{v_l}_{c_j}$. To see this, consider a simple mass–action or power-law rate law without regulation: the elasticities are directly given by the thermodynamic terms

$$\hat{E}^{v_l}_{c_i} = \frac{m^{\rm S}_{li}\,\zeta_l - m^{\rm P}_{li}}{\zeta_l - 1} = \begin{cases} i \text{ is a substrate} & : \ \frac{\zeta_l}{\zeta_l-1}\, m^{\rm S}_{li} \\ i \text{ is a product} & : \ \frac{-1}{\zeta_l-1}\, m^{\rm P}_{li} \end{cases} \tag{S22}$$

$$\hat{E}^{v_l}_{c_i c_j} = -\frac{\zeta_l\, h_l^2\, n_{il}\, n_{jl}}{(\zeta_l - 1)^2} = \begin{cases} i,\, j \text{ are substrates} & : \ -\frac{\zeta_l\, m^{\rm S}_{li}\, m^{\rm S}_{lj}}{(\zeta_l-1)^2} \ \approx \ -\frac{1}{\zeta}\, \hat{E}^{v_l}_{c_i}\, \hat{E}^{v_l}_{c_j} \\ \text{one substrate, one product} & : \ \frac{\zeta_l\, m^{\rm S}_{li}\, m^{\rm P}_{lj}}{(\zeta_l-1)^2} \ \approx \ -\hat{E}^{v_l}_{c_i}\, \hat{E}^{v_l}_{c_j} \\ i,\, j \text{ are products} & : \ -\frac{\zeta_l\, m^{\rm P}_{li}\, m^{\rm P}_{lj}}{(\zeta_l-1)^2} \ \approx \ -\zeta\, \hat{E}^{v_l}_{c_i}\, \hat{E}^{v_l}_{c_j} \end{cases}. \tag{S23}$$

In this formula, the distinction between substrates and product is not made based on the actual flux direction, but on their roles in the reaction formula; the flux direction enters the formulae via ζ_l, which may be larger or smaller than 1, depending on the sign of the thermodynamic force and thus on the flux direction. The second-order elasticities read

$$\hat{E}^{v_l}_{c_i c_j} = \vartheta^l_{ij}\, \hat{E}^{v_l}_{c_i}\, \hat{E}^{v_l}_{c_j}, \tag{S24}$$

where, in the present case (non-regulated mass–action rate law), the prefactor ϑ^l_{ij} reads

$$\vartheta^l_{ij} = \begin{cases} i,\, j \text{ are substrates} & : \ -1/\zeta_l \\ \text{one substrate, one product} & : \ -1 \\ i,\, j \text{ are products} & : \ -\zeta_l \end{cases}. \tag{S25}$$

Due to this negative prefactor, we can expect a negative statistical correlation between the second-order elasticity $\hat{E}^{v_l}_{c_i c_j}$ and the product $\hat{E}^{v_l}_{c_i}\,\hat{E}^{v_l}_{c_j}$ of first-order elasticities. In particular, close to equilibrium (where $\theta_l \approx 0$ and therefore $\zeta_l \approx 1$), we obtain $\vartheta^l_{ij} \approx -1$ and thus the general formula $\hat{E}^{v_l}_{c_i c_j} \approx -\hat{E}^{v_l}_{c_i}\,\hat{E}^{v_l}_{c_j}$, which is symmetric between substrates and products. For completely forward-driven reactions (with $\theta_l \to \infty$ and $\zeta_l \to \infty$), in contrast, we obtain $\hat{E}^{v_l}_{c_i c_j} \approx 0$ because the factor $\zeta_l/(\zeta_l - 1)^2$ is close to 0. Can we expect the same relationship also for other rate laws? For generic saturable rate laws, a splitting as in Equation (S24) is formally possible, but there is no simple formula for ϑ^l_{ij}. Therefore, a tendency for negative correlations between $\hat{E}^{v_l}_{c_i c_j}$ and $\hat{E}^{v_l}_{c_i}\,\hat{E}^{v_l}_{c_j}$ may remain, but the negative correlation will be weaker.

S2.3 Metabolic Control Theory

Steady states and metabolic control A steady state is a metabolic state in which metabolite concentrations and fluxes are constant in time. Steady-state fluxes $v_l(\mathbf{e}, \mathbf{x})$ and concentrations $c_i(\mathbf{e}, \mathbf{x})$ depend on enzyme levels e_l and external metabolite concentrations x_j. These dependencies may be complicated and

not explicitly known (see Figure S2.1 (c)). However, if steady-state concentrations and fluxes are given, their sensitivities to parameter changes can be computed from the elasticities. The sensitivity $R^y_{p_m} = \partial y/\partial p_m$ between a target variable y—e.g., a stationary concentration c_i or a flux v_l—and model parameters p_m is called a response coefficient. If each reaction has one reaction-specific parameter p_l, for example the enzyme level e_l, then we can divide the response coefficients R^{y}_{il} by the elasticities $E^{v_l}_{p_l}$ and obtain the control coefficients $C^y_{v_l} = R^y_{p_l}/E^{v_l}_{p_l}$ (Figure 1 (c) in main text). Control coefficients describe how local perturbations of a reaction rate affect the network-wide steady state. By definition, they depend on the perturbed reaction, but not on the perturbation parameter. Thus, response coefficients refer to perturbed parameters and control coefficients to perturbed reactions. The effects of global parameters such as temperature, which affect many reactions, are described by response coefficients $R^y_{p_m} = \sum_l C^y_{v_l}\, E^{v_l}_{p_m}$ (for more details, see Section S5.2 and [7, 10, 8]). Elasticities, response coefficients, and control coefficients can be defined in their unscaled form $\partial y/\partial x$ (denoted by a bar $\bar{X}$) or in their scaled form $\partial \ln y/\partial \ln x$ (denoted by a hat $\hat{X}$) (see Section S2.2). If an enzyme catalyses a single reaction, the enzyme level appears as a prefactor in the rate law and its scaled response and control coefficients are identical. The summation and connectivity theorems [2], a central finding of Metabolic Control Theory (MCT), entail linear dependencies among the control coefficients along a stationary flux distribution or in the reactions surrounding a common metabolite. Quantities and formulae related to fluctuations in time (such as spectral response coefficients, spectral power density, and variability on different time scales) are described in the Section S4.

Enzyme synergies The synergy effects of enzyme pairs on a flux v (or on some other steady-state variable) can be approximated by second-order response coefficients, called synergy coefficients. Assume that two enzymes are inhibited, thus decreasing their levels e_a and e_b to small values u^*_a and u^*_b, and that this leads to relative flux changes $w_{\mathrm{a}} = v^{\mathrm{a}}/v$, $w_{\mathrm{b}} = v^{\mathrm{b}}/v$ for the single inhibitions and $w_{\mathrm{ab}} = v^{\mathrm{ab}}/v$ for the double inhibition. Based on these numbers, we define the synergy effect

$$\eta^{\mathrm{v}}_{\mathrm{ab}} \quad = \quad \ln \frac{w_{\mathrm{ab}}}{w_{\mathrm{a}} w_{\mathrm{b}}}. \tag{S26}$$

A positive value of $\eta^{\mathrm{v}}_{\mathrm{ab}}$ indicates a buffering synergy ($w_{\mathrm{ab}} > w_{\mathrm{a}}\, w_{\mathrm{b}}$), while a negative value indicates an aggravating synergy ($w_{\mathrm{ab}} < w_{\mathrm{a}}\, w_{\mathrm{b}}$). If $w_{\mathrm{ab}} = w_{\mathrm{a}}\, w_{\mathrm{b}}$, there is no synergy. In a second-order expansion around the unperturbed state, the synergistic effect can be written as (see Section S5.4)

$$\eta^{\mathrm{v}}_{ab} \quad \approx \quad \hat{R}^{\mathrm{y}}_{e_a e_b} \Delta_{\mathrm{r}} \ln e_a \cdot \Delta_{\mathrm{r}} \ln e_b, \tag{S27}$$

so the scaled synergy coefficients $\hat{R}^{\mathrm{y}}_{e_a e_b}$ quantify synergisms between two enzymes. If perturbations and target variables are measured on non-logarithmic scale, the synergistic effects $\eta^{\mathrm{v}}_{\mathrm{ab}} = w_{\mathrm{ab}} - w_{\mathrm{a}} - w_{\mathrm{b}}$ can be approximated by $\eta^{\mathrm{v}} \approx R^{\mathrm{y}}_{e_a e_b} \Delta e_a\, \Delta e_b$ with the unscaled response coefficient $R^{\mathrm{y}}_{e_a e_b}$.

S2.4 Model Construction by STM

Constraints between parameters and variables in kinetic steady-state models A kinetic steady-state model is determined by its network structure, rate laws, state variables, and saturation values: these values define the elasticities and kinetic constants. For a consistent model, these variables have to satisfy a number of constraints, explained by the dependence schema.

- **Wegscheider conditions** Some biochemical quantities, for example the Gibbs free energies of reactions, can be written as differences $\Delta_{\mathrm{r}} x_l$ along reactions, or in vector form $\Delta_{\mathrm{r}}\mathbf{x} = \mathbf{N}^{\mathrm{tot}\,\top}\mathbf{x}$, where $\mathbf{N}^{\mathrm{tot}}$ is the stoichiometric matrix including both internal and external metabolites. Such quantities must obey the Wegscheider condition $\mathbf{K}^{\top}\Delta_{\mathrm{r}}\mathbf{x} = 0$, where $\mathbf{K}$ is a null-space matrix satisfying $\mathbf{N}^{\mathrm{tot}}\,\mathbf{K} = 0$.

Wegscheider conditions must hold, for example, for equilibrium constants ($\ln \mathbf{k}^{\mathrm{eq}} = \mathbf{N}^{\mathrm{tot}\top} \ln \mathbf{c}^{\mathrm{eq}}$), mass–action ratios ($\ln \mathrm{q}^{\mathrm{ma}} = \mathbf{N}^{\mathrm{tot}\top} \ln \mathbf{c}$), and thermodynamic forces ($\mathbf{A} = -\Delta_{\mathrm{r}}\boldsymbol{\mu} = -\mathbf{N}^{\mathrm{tot}\top}\boldsymbol{\mu}$).

- **Haldane relationships** In a chemical equilibrium state, all metabolic fluxes v_l must vanish. If we consider a single reaction in equilibrium, set its rate to zero ($\nu_l(\mathbf{c}^{\mathrm{eq}}, ...) = 0$), and solve for the equilibrium constant, we obtain an equation between equilibrium constant and kinetic constants, the so-called Haldane relationship [11]. For example, for a reversible mass–action law $v_l = k^{\mathrm{cat}}_{+l} a - k^{\mathrm{cat}}_{-l} b$, the Haldane relationship reads $k^{\mathrm{eq}}_l = k^{\mathrm{cat}}_{+l}/k^{\mathrm{cat}}_{-l}$. For the modular rate laws, it reads $k^{\mathrm{eq}}_l = \frac{k^{\mathrm{cat}}_{+l}}{k^{\mathrm{cat}}_{-l}} \prod_i (k^{\mathrm{M}}_{li})^{h_l\, n_{il}}$ (see [1]).

- **Equilibrium constant and chemical standard potentials** In chemical equilibrium, the Gibbs free energies of reaction $\Delta_{\mathrm{r}} G_l = \Delta_{\mathrm{r}}\mu_l$ must vanish. With the formula for chemical potentials $\mu_i = \mu_i^{\circ} + RT \ln c_i$ (i.e., assuming an activity coefficient and a standard concentration equal to 1), this leads to the formulae $\ln k^{\mathrm{eq}}_l = -\frac{1}{RT}\sum_i \mu_i^{\circ} n_{il}$ and $\theta_l = -\frac{1}{RT}\Delta_{\mathrm{r}}\mu_l = \ln \frac{k^{\mathrm{eq}}_l}{q^{\mathrm{ma}}_l}$.

- **Signs of fluxes and thermodynamic forces** According to the second law of thermodynamics, all chemical reactions must dissipate Gibbs free energy. This implies that rates and thermodynamic forces have the same signs ($v > 0 \Rightarrow \theta > 0$ and $v < 0 \Rightarrow \theta < 0$), in agreement with the relationship $\theta = \ln \frac{v_+}{v_-}$. A stricter version of this constraint, excluding near-equilibrium reactions, imposes a minimum force $|\theta| > \theta^{\mathrm{min}}$.

- **Steady-state fluxes** For applying MCT, the metabolic reference state must be a steady state, i.e., a state in which the metabolic fluxes satisfy the stationarity condition $\mathbf{N}\,\mathbf{v} = 0$. In addition, we may impose bounds $\mathbf{v}^{\mathrm{min}} \leq \mathbf{v} \leq \mathbf{v}^{\mathrm{max}}$ on the reaction rates and bounds $\mathbf{v}^{\mathrm{min}}_{\mathrm{ext}} \leq \mathbf{N}^{\mathrm{ext}}\mathbf{v} \leq \mathbf{v}^{\mathrm{max}}_{\mathrm{ext}}$ on the production or consumption of external metabolites. Such bounds can be used to predefine reaction directions or to keep fluxes close to measured values.

- **Stability of the steady state** For applying MCT to a metabolic reference state, this state must be asymptotically stable, i.e., its Jacobian matrix must not have eigenvalues with positive real parts. This constraint depends on all model details, and we cannot guarantee it by the dependence schema. Following [9], stable states can be obtained by generating a model ensemble and omitting all model instances with unstable states.

- **Amounts or concentrations** In compartmentalised models, we need to distinguish between metabolite amounts and metabolite concentrations, which are related by compartment volumes. Generally, mass balances concern amounts while rate laws depend on concentrations. In STM, fortunately, amounts as such do not play a role. While concentrations appear as model variables, amounts are modelled only implicitly (e.g., if stationarity is imposed on fluxes).

A model construction algorithm that violates these constraints would lead to inconsistent models or metabolic states.

Model construction algorithm In a model with predefined kinetic constants, enzyme levels, and external metabolite concentrations, steady-state fluxes and concentrations can be determined numerically (Figure S2 (a)). However, to construct models with physiologically plausible states, it may be safer to start from reasonable metabolic fluxes and to construct metabolite concentrations and kinetic rate laws around them in such a way that they yield the predefined fluxes (Figure S2 (b)). Model construction based on STM combines ideas from thermodynamic flux analysis [12, 13, 14] (in the metabolic state phase), SKM [9] and thermodynamically consistent model parametrisation [15, 3] (in the kinetics phase). Like in SKM, steady-state concentrations and fluxes can be predefined. To satisfy Wegscheider conditions, Haldane relationships, and the sign constraint between reaction rates and thermodynamic forces, steady state and kinetic constants

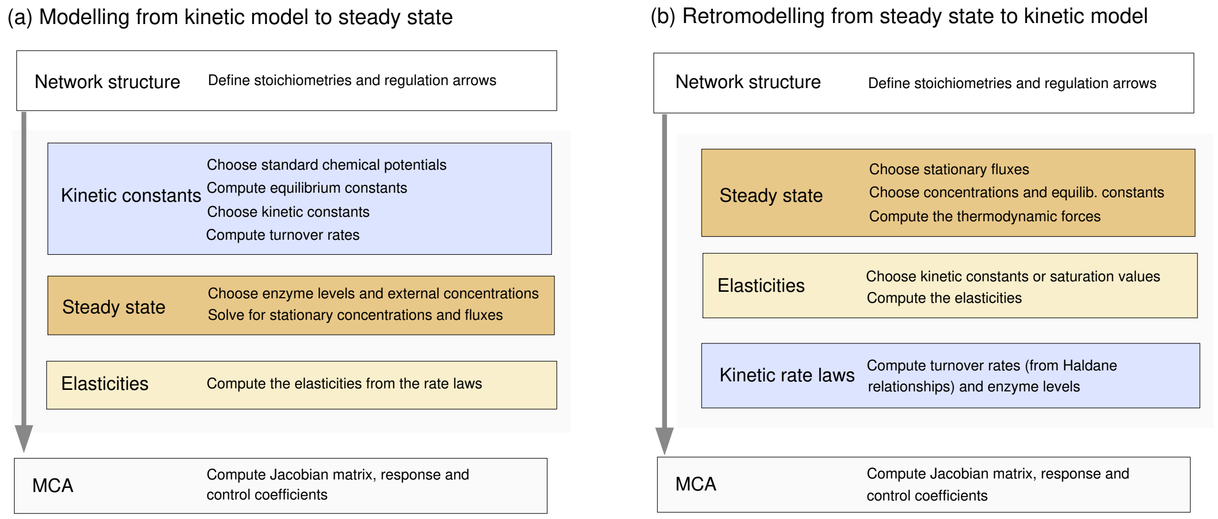

Figure S2: Constructing kinetic metabolic models in steady state. (a) In "causal" kinetic modelling, we start with rate equations and determine a steady state. A kinetic model is defined by network structure and rate laws. Given the kinetic constants, we solve for a steady state and compute the elasticities in this state by taking the derivatives of the rate laws. The elasticities determine the linearised dynamics around our steady state and are central to Metabolic Control Theory. (b) Retromodelling, starting from metabolic fluxes. First, the steady-state variables (metabolite concentrations, fluxes, and equilibrium constants) are chosen under thermodynamic constraints. Then, saturation values or dissociation constants are chosen, and kinetic constants and elasticities are computed. The basic model variables (state variables and saturation values or dissociation constants) can be independently chosen, sampled, or optimised based on predefined values, bounds, or probability distributions. Prior knowledge about kinetic constants can be employed when choosing the saturation values.

are chosen sequentially. The network structure, our starting point, is defined by stoichiometric matrix, regulation matrix, and the list of external metabolites. In the algorithm, basic variables (steady-state variables and saturation values) are determined step by step based on known values, constraints, or probability distributions: they can be chosen manually, by optimisation, by fitting them to data, or by random sampling. Derived variables (e.g., kinetic constants) are computed from variables chosen previously, as described by the dependence schema in Figure 2 (main text). Finally, models can be checked for a stable steady state (if such a state is required for the further analysis, e.g., Metabolic Control Analysis). All models shown in the article passed this test.

Reconstruction of kinetic rate laws In STM, the scaled elasticities are not directly given by saturation values, but depend on saturation values and thermodynamic forces. Each sampled elasticity matrix corresponds to one instance of the model ensemble, that is, zo a particular kinetic model with consistent kinetic constants. In a variant of the STM algorithm, we do not sample the saturation values directly ($\beta^{\rm M}_{li}$, $\beta^{\rm A}_{li}$, $\beta^{\rm I}_{li}$), but compute them from sampled dissociation constants ($k^{\rm M}_{li}, k^{\rm A}_{li}$, and $k^{\rm I}_{li}$). In both cases, the catalytic constants $k^{\rm cat}_{\pm}$ are computed from two constraints: the ratio $k^{\rm cat}_{+l}/k^{\rm cat}_{-l}$ is determined by the Haldane relationships while their geometric mean $\sqrt{k^{\rm cat}_{+l} \cdot k^{\rm cat}_{-l}}$, treated as a model parameter $k^{\rm V}$, depends on their absolute scaling. To choose this scaling, we may either predefine the enzyme levels e_l and scale the catalytic constants $k^{\rm cat}_{\pm}$ such that the reaction rate matches the predefined flux; or we predefine the $k^{\rm cat}_{\pm}$ values and solve for the enzyme levels.

Combining modular and hand-curated rate laws Once generic rate laws have been assigned, some of them may be replaced by more detailed rate laws (obtained from enzyme assays) to make the model more realistic [16]. To ensure a consistent model, the fluxes, concentrations, and equilibrium constants in these

reactions must be equal between the reconstructed model and the new rate laws to be inserted. There are different ways to guarantee this: either reaction rates and equilibrium constant, taken from the rate law, are imposed as constraints when sampling the elasticities; or kinetic constants and enzyme level in the rate law are adjusted to the network model[3].

S3 Model Ensembles

S3.1 Sampling the Model Variables

Model ensembles Our models represent samples from an ideal, infinitely large model ensemble defined by three types of information: (i) restrictions on model structure and steady state, set by the modeller (e.g., the choice of a fixed flux distribution); (ii) the independence between basic variables, and dependencies of other variables on them, as encoded in the dependence schema (Figure 2 in main text); (iii) the random distributions from which the basic variables are sampled. Together, these choices define the distributions and statistical dependencies of all model variables. Any kinetic model that satisfies the constraints can be obtained by the algorithm.

Standard saturation values of $\frac{1}{2}$ Why are saturation values of $\frac{1}{2}$ a meaningful choice (assuming substrate concentrations $c = k^{\mathrm{M}}$)? In an irreversible Michaelis–Menten rate law, an enzyme with a scaled substrate elasticity of $\frac{1}{2}$ would work at its half-maximal rate. To see this, we note that $\frac{\mathrm{d}}{\mathrm{d}x}\frac{1}{1+x} = \frac{1}{(1+x)^2}$: in the point $x = 1$, the function value is $\frac{1}{2}$ and the value of the derivative is $1/4$, so the scaled elasticity in this point is given by $\frac{1/4}{1/2} = \frac{1}{2}$. In our reversible modular rate laws, k^{M} denotes the dissociation constant (the concentration leading to half-saturation). A concentration equal to the k^{M} value need not lead to a half-maximal rate, but it still represents a good compromise between a very sensitive but inefficient enzyme (in the linear range) and a very efficient but insensitive enzyme (at full saturation).

Beta distributions In the sampling of saturation values, what are the reasons for using beta distributions? The beta distribution is a probability distribution on the interval [0, 1], with two parameters determining the mean and width. It generalises the uniform distribution and provides a convenient description for saturation values for several reasons. A saturation value represents a concentration and corresponding dissociation constant, combined in the form $X/(X+Y)$. If two independent random variables X and Y follow gamma distributions with the same scale parameter, the random variable $X/(X+Y)$ will follow a beta distribution. Gamma distributions with suitable shape parameters resemble log-normal distributions, which have been previously used for choosing concentrations and dissociation constants, for example in parameter balancing [17]. The log-normal distributions of biological variables represent the assumption that the variables are positive and determined by many independent, multiplicative random effects. A second reason for using beta distributions is that saturation values represent the occupancy of enzyme binding sites. At given enzyme molecule number and occupation probabilities, the count numbers of occupied binding sites are binomially distributed. The beta distribution is the conjugate prior of this distribution. Therefore, in a Bayesian model for binding site occupancies, it is a natural choice for describing priors and likelihood terms for occupation probabilities.

Sampling basic variables randomly or based on data In the metabolic state phase, a flux distribution may be chosen by flux minimisation. Metabolite concentrations, Gibbs free energies of formation, and thermodynamic forces may be determined by parameter balancing [17], using upper and lower bounds for concentrations and thermodynamic forces, known values (for concentrations) and predicted values (for Gibbs

[3]If an irreversible rate law is given, the equilibrium constant can be ignored in the network model

free energies of formation) as data, and flux directions as inequality constraints. Upper and lower bounds, signs, predefined values, and distributions used for sampling reflect model assumptions and available data; by choosing them, we can adjust the model to specific metabolic states and to kinetic or metabolic data. Fluxes and thermodynamic forces may be sampled uniformly, under linear constraints and with predefined sign patterns. metabolite concentrations, enzyme levels, and dissociation constants can be sampled from log-normal or gamma distributions, and saturation values can be sampled, e.g., from uniform or beta distributions. In all phases of model construction, instead of sampling the variables freely, experimental data can be inserted, or distributions centred around data values can be used. To use experimental data in a more solid way, the entire model construction procedure can also be integrated into a Bayesian framework, in which a posterior for the model parameters is determined from data and priors.

Varying the thermodynamic forces at given fluxes To systematically study the impact of thermodynamic forces on model dynamics, one may vary the thermodynamic forces at a fixed metabolic flux distribution. Varying the thermodynamic forces (e.g., doubling all their values) requires a variation of metabolite concentrations, but these metabolite variations are not uniquely defined. For a simple procedure, we start from a metabolic state with concentration vector $\mathbf{c}^{\rm orig}$ and force vector $\boldsymbol{\theta}^{\rm orig}$ (which must agree with the flux directions). To realise a different force vector $\boldsymbol{\theta}$ (with the same signs as $\boldsymbol{\theta}^{\rm orig}$), we choose the new concentration vector

$$\begin{aligned} \mathbf{c} &= \mathrm{argmin}_{\mathbf{c}}\, ||\ln \mathbf{c} - \ln \mathbf{c}^{\rm orig}||^2 \\ \text{s.t.}\quad \boldsymbol{\theta} - \boldsymbol{\theta}^{\rm orig} &= -\mathbf{N}^\top [\ln \mathbf{c} - \ln \mathbf{c}^{\rm orig}]. \end{aligned} \tag{S28}$$

The idea is to apply a minimal change in the metabolite profile (Euclidean distance on logarithmic scale). In this procedure, we may impose upper and lower bounds on $\mathbf{c}$. However, depending on the bounds there may be no solution.

S3.2 Do Model Variants Differ in Their Behaviour? Some Useful Statistical Tests

Among the dynamic features of a metabolic model, which of them result from network structure and which depend mostly on quantitative factors like kinetic constants? To pose this question more generally, we may consider two model variants that differ in some aspect (e.g., network structure or flux distribution), while other aspects (e.g., rate constants) can be varied for each of the variants. Then, we ask whether a (qualitative or quantitative) model output differs significantly between the variants. To prove or disprove such differences, we describe each model variant by a model ensemble, sample instances from both ensembles, compute their target variables, and compare them for significant differences. This allows us, for example, to compare two variants of a kinetic model and to check whether these differences between the variants lead to typical differences in synergy patterns, irrespective of variable kinetic parameters. The test described below has been implemented in Matlab (see github.com/liebermeister/stm).

Significant differences in binary target variables If we consider a qualitative model property (e.g., is the steady state stable or unstable?), each model ensemble can be characterised by the fraction p of "positive" model instances. Given a set of sampled model instances (with count numbers n_+ and n_- for "positive" and "negative" model instances), the true fraction p can be estimated by Bayesian estimation. If p were the true fraction, the number n_+ of positive model instances (out of $N = n_+ + n_-$ model instances sampled) would be binomially distributed with mean $p\,N$ and maximal value N. Knowing this, we can estimate the value of p from the given number n_+ by using Bayesian estimation. Assuming a flat prior, the posterior of p is a beta distribution $\mathrm{Prob}(p) \sim p^{\alpha-1}(1-p)^{\beta-1}$, where $\alpha = n_+ + 1$ and $\beta = N - n_+ + 1$. The mean value of this

distribution, $\langle p \rangle = \alpha/(\alpha+\beta) = (n_+ + 1)/(N+2)$, can be used as an estimator for p. The corresponding variance reads $\sigma_p = \frac{\alpha\beta}{(\alpha+\beta)^2\,(\alpha+\beta+1)} = \frac{(n_+ +1)(N-n_+ +1)}{(n_{\mathrm{perm}}+2)^2\,(N+3)}$.

Significant differences in the occurrence of positive and negative synergies Given a matrix of enzyme synergies (where small-magnitude values have been removed by thresholding), we count the positive and negative synergies (numbers n_+ and n_-) between two groups of enzymes, e.g., between enzymes involved in two metabolic pathways. As a null hypothesis, we assume that synergies can be positive or negative with equal probabilities. Under this null hypothesis, and assuming that only very few synergies remain after thresholding, n_+ and n_- would be independently binomially distributed with the same unknown mean value n. Given n, the difference $n_+ - n_-$ would have a mean value of 0 and a standard deviation of $\sqrt{2\,n}$. Thus, the ratio between an observed difference $n_+ - n_-$ and this standard deviation can be used as a score for sign bias. Since the value of n is unknown, we approximate it by $\frac{n_+ + n_-}{2}$ and obtain the empirical sign bias score $z_{\mathrm{sign}} = \frac{n_+ - n_-}{\sqrt{n_+ + n_-}}$.

Quantitative target variables Finally, we consider quantitative target variables and their differences between model variants as seen in model ensembles. As an example, we consider predicted enzyme synergies. The general idea is as follows. To see whether a synergy (between two enzymes i and j) differs significantly between two model variants, we compute the synergies for many instances of the two model variants, take the mean value for each variant, and compare the two values using a p-value (obtained from a permutation test) as a criterion for significant differences. Since we run many such significance tests in parallel (namely, for many different enzyme pairs), we expect a certain amount of false positives. To account for multiple testing, we choose a false discovery rate and select significant enzyme pairs based on their p-values [18]. This is how the procedure works in detail:

1. **Sample synergy values** We sample n_{model} model instances for each of the two model variants. For each model instance, we compute the synergies of all enzyme pairs. Altogether, we obtain a collection of synergy values η_{ijk}, indexed by $i \in 1,..,n_{\mathrm{pairs}}$ for enzyme pairs, $j \in \{1,2\}$ for the two model variants, and $k \in 1,..,n_{\mathrm{inst}}$ for the sampled model instances of each variant. If the network contains n_{r} enzymes, there are $n_{\mathrm{pairs}} = n_{\mathrm{r}}(n_{\mathrm{r}}-1)/2$ enzyme pairs, i.e. possible synergies, to be computed. The synergy data η_{ijk} are now tested for significant differences.

2. **Quantify large (positive or negative) synergies by p-values** For each enzyme pair i, we first test whether this pair shows a significantly large (or small) synergy value caused by the model structure. If it does, the synergy values will stand out from the general distribution of synergy values, even if we average over many random choices of the kinetic parameters. We apply the following statistical test: for each enzyme pair i, we test whether the mean value $\eta_{i\cdot\cdot}$ (averaged over all model variants and all Monte Carlo samples) is significantly larger (or smaller) than other mean synergy values. We use a permutation test: the actual mean value $\eta_{i\cdot\cdot}$ for our enzyme pair is compared to mean values obtained from batches of resampled η_{ijk} values. In each permutation run $l \in 1,..,N$, we resample $2 \cdot n_{\mathrm{inst}}$ of the η_{ijk} values with replacement and compute their mean value $\langle \eta_{il} \rangle$. Let n_i denote the number of resampled mean values $\langle \eta_{il} \rangle$ larger than $\eta_{i\cdot\cdot}$. Whether $\eta_{i\cdot\cdot}$ is significantly large is indicated by a p-value p_i, estimated by

$$p_i = \frac{n_i + 1}{N+2} \tag{S29}$$

(for a justification, see the above treatment of binary variables). Small values $p_i \approx 0$ indicate that $\eta_{i\cdot\cdot}$ is larger than expected by chance (i.e., significantly large), large values $p_i \approx 1$ indicate that $\eta_{i\cdot\cdot}$ is smaller than expected by chance (i.e., significantly small).

3. **Quantify differences in synergies (between model variants) by p-values** Next, for each enzyme pair i, we ask whether the synergy values differ significantly between the two model variants. In the test, we consider the mean values $\eta_{i1\cdot}$ and $\eta_{i2\cdot}$ of the two model variants, averaged over all kinetic parameter samples, and check whether they differ significantly. Again, we use a permutation test. This time, we compute the mean difference $\Delta\eta_{ij} = \eta_{i1\cdot} - \eta_{i2\cdot}$. In each run d of the permutation test, we randomly permute the values η_{ijk} for the pair a under study, divide them into two batches of size n_{inst}, and compute the mean difference $\Delta\langle\eta_{il}\rangle$ between the two batches. A p-value, stating whether $\Delta\eta_{ij}$ is large, is computed as above by counting how many of the permutation samples lead to larger values.

4. **Select significant synergies or differences based on p-values** Given the previously computed p-values, we determine which of their mean values $\eta_{\cdot j\cdot}$ and of their mean differences $\Delta\eta_{\cdot j\cdot}$ are significantly high (or low). Since we test this for many n_{pairs} gene pairs, we need to account for multiple testing: we fix a false discovery rate of 5% and choose the confidence level $\alpha = 0.05$ for the individual tests. With this choice, $\alpha \cdot n_{\mathrm{pairs}}$ of the apparently significant values (for each of the four tests, high or low mean synergy and difference in synergy) are expected to be false positives.

S3.3 Extensions of STM

The algorithm for model construction can be extended in many ways:

- **Cell compartments** In kinetic models with compartments, the compartment sizes appear in the balance equations and may follow differential equations themselves. In our model construction, compartments are not modelled explicitly, but a compartment structure can be added to the reconstructed kinetic model. This, however, changes control properties such as Jacobian, response, and control matrices.

- **Dilution by cell growth** In models with dilution (due to growth rate λ), intracellular metabolites will be effectively "consumed" by a dilution flux $\lambda\, c_i$. This changes the stationarity condition and directly couples fluxes to metabolite concentrations. Given a steady state, the elasticities can be computed as normally, but the Jacobian, response, and control coefficients will be affected. The elasticities for the dilution reactions are directly given by dilution rate and metabolite concentrations.

- **Thermodynamically infeasible fluxes** Equation (2), a main formula in elasticity sampling, requires that flux directions follow the thermodynamic forces, that is, fluxes must lead from higher to lower chemical potentials. In practice, even valid flux distributions may violate this assumption, for example, if cofactors or protons are omitted in the model. To apply STM regardless, we may choose to ignore thermodynamics and treat some reactions as irreversible—that is, we ignore the thermodynamic term in the substrate elasticity and set the product elasticity to zero. Alternatively, we may adjust the thermodynamic forces to the given flux directions: whenever $\mathrm{sign}(-\Delta_{\mathrm{r}}\mu_l)$ differs from the flux direction, we add a virtual substrate and choose its chemical potential μ_x such that the thermodynamic force $-\Delta_{\mathrm{r}}\mu^* = -(\Delta_{\mathrm{r}}\mu_l + \mu_x)$ has the correct sign. The virtual substrate changes the equilibrium constants and thermodynamic forces, but can otherwise be ignored in the kinetic rate law.

- **Avoiding divergencies close to chemical equilibrium** In reactions close to chemical equilibrium, with a thermodynamic force $\theta_l \approx 0$, the one-way rates $v_l^{\pm}$ and scaled elasticities become very large. In modelling, this may cause numerical problems. Moreover, also implies that the enzyme is very fast or abundant, and thus able to sustain a finite flux at an enzyme efficiency close to 0. To avoid such unrealistic assumptions our models, we set a constraint $v_l^{\pm} < \rho\,|v_l|$ on the forward and backward rates

in each reaction. With a threshold $\rho = 100$, for example, forward fluxes can be at most 100 times as large as the net flux. This translates into a constraint for thermodynamic forces in the metabolic state phase: the flux sign constraint ($v_l \neq 0 \Rightarrow \mathrm{sign}(v_l)\,\theta_l \geq 0$) is replaced by the stricter constraint[4] $v_l \neq 0 \Rightarrow \mathrm{sign}(v_l)\,\theta_l \geq 1/\rho$. It will prevent extreme values in the thermodynamic elasticity term $\hat{E}^{\mathrm{rev}}_{li}$. The unscaled elasticities, in contrast, do not diverge in chemical equilibrium and can be computed from non-divergent formulae [1].

- **Enzyme reactions composed of elementary steps** If we think of enzyme mechanisms as composed of elementary mass–action steps, we can also represent them in this way in a model, replacing each reaction by a more fine-grained description. In the resulting model, there is a much larger number of (elementary) reactions. Since all rate laws are mass–action kinetics, the elasticities are directly given by reversibility terms and completely determined by thermodynamic forces. It sounds surprising: by knowing the fluxes and thermodynamic forces, we completely know the enzyme kinetics! But we should not forget that we are now talking about the thermodynamic forces of *elementary steps* and that the equilibrium constants of these steps corresponds to k^{M} values (of saturable modular rate laws) in the more coarse-grained model.

- **Prior distributions for saturation values** Saturation values $\beta = k/(k + x)$ can be set to fixed values (e.g., $\beta = 0$ for enzymes believed to be in the linear range, $\beta = \frac{1}{2}$ for enzymes in half-saturation, or $\beta = 1$ for enzymes in full saturation), or they can be sampled independently from the range $]0, 1[$. If β is drawn from a uniform distribution (as suggested by the principle of minimal information [19]) and if the metabolite concentrations are fixed, the resulting dissociation constant k is randomly distributed with probability density $p(k) = \frac{k}{(c+k)^2}$ (see Section S5.1). If a saturation value is approximately known, we can use a beta distribution instead, with density $p(\beta) \sim \beta^{a-1}(1-\beta)^{b-1}$ with a mean value $a/(a+b)$ given by the known value. This yields a distribution $p(k) = \frac{k}{(c+k)^2}(\frac{c}{k+c})^{a-1}(\frac{k}{k+c})^{b-1} = \frac{k^{b-1}c^{a-1}}{(k+c)^{a+b}}$ for the dissociation constant. Saturation values can also be sampled from dependent distributions: this may be necessary if enzymes bind to different reactants, for example NAD^+ and NADH, with unknown but similar binding affinities, leading to correlations between their saturation values in physiological states.

- **Multiple steady states** Under different choices of external metabolite concentrations and enzyme levels, a kinetic model will show different steady states. Each of these states is characterised by different metabolite concentrations, fluxes, saturation values, and elasticities. For constructing a model with multiple states directly, the STM algorithm needs to be modified: we need to ensure that the saturation values and elasticities in different states correspond to the same set of kinetic constants. This is simple. In the metabolic state phase, we sample a set of equilibrium constants and different sets of state variables for the multiple steady states; in the kinetics phase, we sample a single set of kinetic constants, which determine the saturation values, elasticities, and enzyme levels for each steady state. Again, enzyme levels are determined last; to adjust them to measured enzyme levels from proteomics, some previously chosen model variables can be adjusted (e.g. the velocity constants). Another possibility to account for enzyme data is to embed the entire algorithm into another layer of parameter fitting or posterior sampling.

- **Adaptation of enzyme levels** In our metabolic models, enzyme levels appear as parameters. In reality, they are controlled by transcriptional regulation, an important mechanism for shaping metabolic behaviour. To include transcriptional regulation into our metabolic models, the model must be extended to describe the production of enzymes. Alternatively, we may treat enzyme levels as choice

[4]The constraint can be derived as follows (assuming $v_l > 0$ without loss of generality): the ratio between forward and net reaction rate is given by $v_{+l}/v_l = \zeta_l/(\zeta_l - 1)$. Close to equilibrium, we can approximate $1/\zeta_l \approx 1 - \theta_l$ and obtain $\rho \geq \frac{v_{+l}}{v_l} = \frac{1}{1-1/\zeta_l} \approx 1/\theta_l$.

variables and attempt to derive plausible enzyme adaptation profiles from optimality considerations [20]. To improve metabolic efficiency, enzymes should be expressed in the right proportions to adapt continuously to the current metabolic tasks. For instance, a rising demand for a certain metabolite may lead to the induction of a biosynthesis pathway for this metabolite. If such feedback systems are in place, changes in enzyme levels will affect the metabolic state, and induce secondary adaptations of other enzymes, and so on. In order to predict the effects of, e.g., gene knock-downs, we need to consider the global interplay of such adaptations. Given a kinetic model with a metabolic objective function and an enzyme cost function, optimal enzyme adaptations to external changes or single-enzyme knock-downs can be predicted with the help of synergy coefficients [20]. Again, the predicted enzyme adaptations reflect network structure and elasticities.

- **Analysis of sampled target variables** A model ensemble can be seen as a statistical model with independent ("basic") and dependent ("derived") variables. The dependencies are described by a schema like the one from Figure 2 (main text). Each binary property (e.g., the sign of a control coefficient) has a certain probability in the ensemble. In practice, we can only estimate this probability from a limited number of model instances. If n out of N sampled models show the property P, the probability of P, called q, can be estimated as follows: assuming that q has a uniform prior, its posterior mean and variance read $\mu_q = \frac{n+1}{N+2}$ and $\sigma_q^2 = \frac{(n+1)(N-n+1)}{(N+2)^2(N+3)}$. For each target variable (e.g., a control coefficient), we obtain a number of sampled values. Their distribution can be characterised by mean value, variance, probabilities of signs, and correlations with other model variables.

- **Significant differences between model variants** By choosing network structure, fluxes, thermodynamic forces, and enzyme saturation step by step, we can implement a nested sampling procedure that produces a hierarchy of model variants. In this hierarchy, each model variant is represented by a model ensemble with a specific distribution of target variables. To make model variants comparable, the variants should at least have the same metabolite and reaction lists, but they can differ in their network structures and in the values assigned to any of the basic variables. Using statistical tests, we can determine significant differences between the distributions of model targets, if necessary with corrections for multiple testing (see Section S3.2 for details). By comparing the distributions of model variables between subensembles, we can study how structural model features affect the target variables: for example, whether certain regulation arrows can enhance the stability of steady states. More generally, we can systematically study the effects of network structure, regulation, thermodynamic forces, enzyme saturation, and different rate laws on our model outputs.

- **Choosing the distributions of target variables** To see how specific values or ranges of target variables can be obtained, we can build a model ensemble and then filter it for models that show these values or ranges. Again, we obtain a subensemble of models with different distributions and correlations of the model variables. Even basic variables that were chosen independently can become dependent by the subselection. Alternatively, we can set the distribution of our target variables during model construction by applying a Bayesian posterior sampling. In this approach, the basic variables are not sampled freely but by a Metropolis Monte Carlo procedure: as prior distributions, we can use the same probability distributions as employed normally in STM; for the likelihood function, we compare the resulting target variables to the prescribed distribution, for example, a distribution defined by experimental data.

S3.4 Example Model: Glycolysis in Human Hepatocytes

In the original publication [21], thermodynamically feasible fluxes were determined by flux minimisation with various different flux objectives. I first focused on aerobic rephosphorylation of ATP on glucose. Optimising

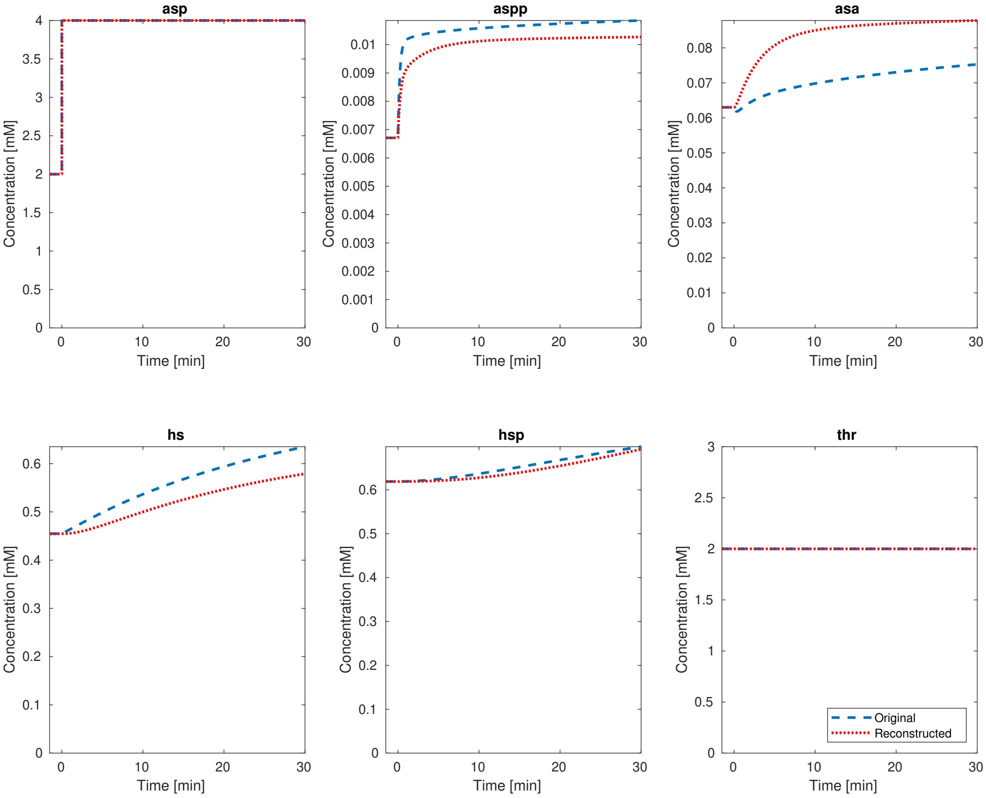

Figure S3: A kinetic model reconstructed by STM. The threonine synthesis pathway in *E. coli* converts aspartate into threonine. A kinetic model from [22] was used to simulate the metabolic dynamics after a sudden increase of the external aspartate level (dashed blue lines): the internal metabolite concentrations increase with different time delays. Aspartate and threonine are treated as external metabolites with predefined concentrations. Based on the network structure, steady state fluxes, and concentrations as in the original model, a model was reconstructed by STM. The reconstructed model shows the qualitative behaviour (solid red lines). Abbreviations: asp (aspartate); aspp (aspartyl phosphate); asa (aspartate beta-semialdehyde)); hs (homoserine); hsp (O-phospho-homoserine); and thr (threonine).

this objective leads to a sparse flux distribution that uses only a small part of the network, containing glycolysis and TCA cycle. With this flux distribution, I first determined a standard model in which all saturation values were set to values of $\frac{1}{2}$, assuming half-saturated enzymes. In the resulting state, the steady ATP rate (our model output) is strongly controlled by glucose import; all control coefficients are positive, i.e., a small increase of any enzyme will always increase the metabolic target (ATP level). Although the flux distribution was chosen to support ATP rephosphorylation, this is not a trivial finding: first, flux analysis can capture the ATP rephosphorylation rate, but not the ATP level as a target function; second, flux analysis describes which fluxes—and in which proportions—are optimal to realise a certain metabolic objective, but it does not capture the marginal effects of enzyme levels, i.e., how the objective would change following small enzyme changes. By sampling the saturation values, we obtain a model ensemble, and the statistical distribution and correlations of these control coefficients can be studied.

S4 Metabolic Synergies and Fluctuations

S4.1 Synergies Between Static Perturbations

Synergy effects between enzymes can be important in medical applications, e.g., to model drug interactions or patient-specific side effects, to plan combination therapies, and to prevent bacterial resistance [23]. Epis-

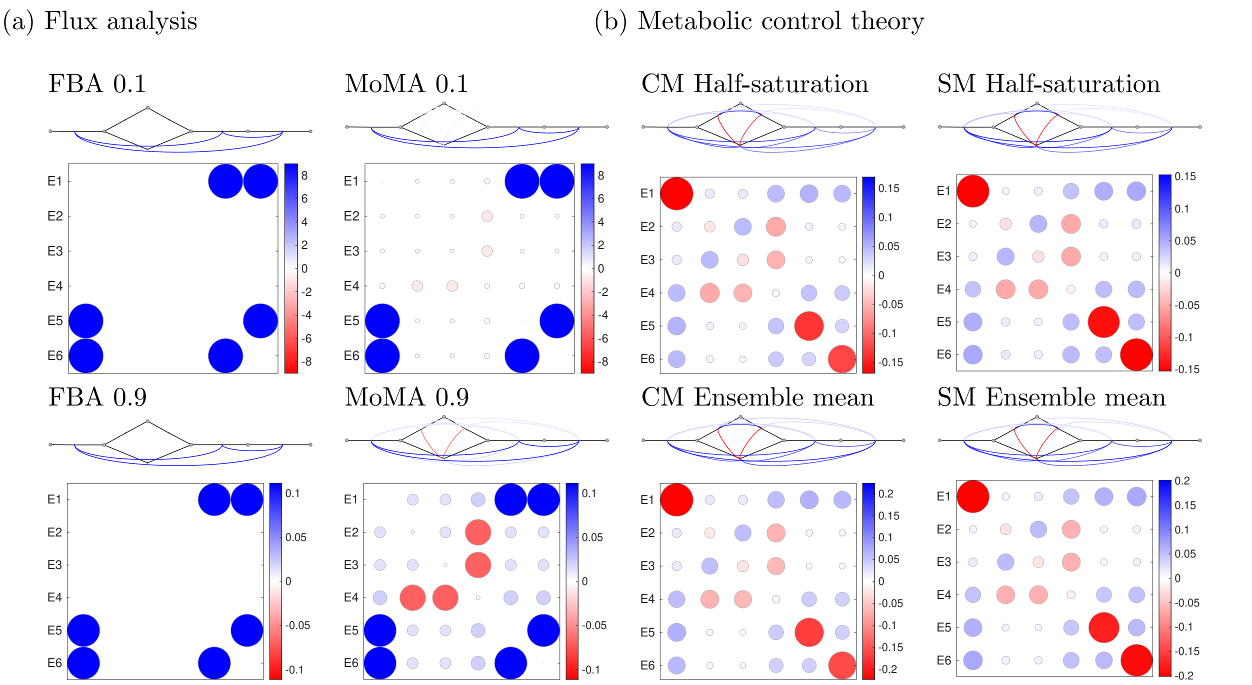

Figure S4: Enzyme synergies in a schematic metabolic pathway. This figure is an extended version of Figure 5 (main text). Enzyme synergies in a linear pathway with alternative routes between the intermediates S1 and S3. (a) Synergies for double inhibitions, predicted by constraint-based methods. Synergies are shown by arc colours (red: aggravating, blue: buffering). Different panels show synergies computed by different methods (FBA and MoMA) and inhibition strengths (flux decrease by applying scaling factors of 0.1 or 0.9). In the calculation, the "double inhibition" of a single enzyme is realised by applying the relative inhibition twice, i.e., leading to inhibition strengths of 0.01 and 0.81, respectively. Colour scales differ between panels, spanning the range of synergy values in each case. Small values (below one percent of the maximal absolute value) are not shown. (b) Synergies computed by Metabolic Control Theory. The panels show results for different rate laws (CM: common saturable rate law; SM: simultaneous-binding modular rate law). Results based on half-saturated enzymes (top) are compared to mean results from a model ensemble with random saturation values (bottom). The two results are almost identical.

tasis, an important concept in genetics, denotes the synergistic effects of gene knock-outs on cell viability. Epistasis can shape genetic variability in populations and the evolvability of genetic features. Moreover, as shown by FBA simulations and experimentally, epistatic interactions can indicate functional associations between proteins, for example the cooperation or alternative usage of enzymes in metabolic pathways [24]. Importantly, while synergies are described here for enzyme perturbations, they can also be computed and used for any other parameter perturbations, including synergistic effects between concentrations in the growth medium.

Epistasis Epistasis describes synergy effects of gene deletions on Darwinian fitness. In buffering epistasis (that is, the effect of a double deletion is less severe than expected), the loss of a first gene decreases the selection pressure on the second one: in evolution, such genes will tend to co-occur in genomes, a phenomenon called phylogenetic correlation [25]. In the opposite case, called aggravating epistasis, the double-deletion phenotype is more severe than expected: here the loss of one gene increases the selection pressure on the other one, leading to phylogenetic anti-correlation. In [24], epistatic synergies in the yeast *S. cerevisiae* were computed by FBA. The maximal biomass production rate was used as a quantitative output function and enzyme deletions were simulated by setting the corresponding reaction rates to zero. The predicted epistasis pattern showed a modular structure [24].

Epistasis measure by Segrè *et al.* Segrè *et al.* introduced a synergy score for double enzyme deletions [24], in which a special weighting makes buffering synergies better detectable. Let v_{wt} denote a target variable observed in the wildtype network (e.g., maximal biomass production rate computed by FBA), and let v_{a}, v_{b}, and v_{ab} denote the values in mutant networks in which enzyme a, enzyme b, or both, have been deleted. After a scaling by the wild-type value, the target values read $w_{\mathrm{wt}} = 1$, $w_{\mathrm{a}} = v_{\mathrm{a}}/v_{\mathrm{wt}}$, $w_{\mathrm{b}} = v_{\mathrm{b}}/v_{\mathrm{wt}}$, and $w_{\mathrm{ab}} = v_{\mathrm{ab}}/v_{\mathrm{wt}}$. Since deletions can decrease, but cannot increase the target in FBA, the values must satisfy $w_{\mathrm{a}} \leq 1$, $w_{\mathrm{b}} \leq 1$, and $w_{\mathrm{ab}} \leq \min(w_{\mathrm{a}}, w_{\mathrm{b}})$. The effect w_{ab} of a double deletion is compared to the effects w_{a} and w_{b} of single deletions, yielding an epistasis score. To obtain a clear distinction between neutral ($w_{\mathrm{ab}} = w_{\mathrm{a}}\, w_{\mathrm{b}}$), aggravating ($w_{\mathrm{ab}} < w_{\mathrm{a}}\, w_{\mathrm{b}}$), and buffering ($w_{\mathrm{ab}} > w_{\mathrm{a}}\, w_{\mathrm{b}}$) gene pairs, Segrè *et al.* introduced a heuristic epistasis measure with the following definition:

$$\varepsilon_{\mathrm{ab}}^{\mathrm{Segre}} = \begin{cases} \text{neutral} & : \ 0 \\ \text{aggravating} & : \ \frac{w_{\mathrm{ab}}}{w_{\mathrm{a}}\, w_{\mathrm{b}}} - 1 \\ \text{buffering} & : \ \frac{\frac{w_{\mathrm{ab}}}{w_{\mathrm{a}}\, w_{\mathrm{b}}} - 1}{\frac{1}{w_{\mathrm{b}}} - 1} \end{cases} . \tag{S30}$$

In the formula for buffering epistasis, we assume $w_{\mathrm{a}} \leq w_{\mathrm{b}}$ without loss of generality. In summary, neutral and aggravating synergies are defined “normally”, but buffering synergies are given special weights: if one of the single-deletion effects is mild, then w_{b} is close to 1 and the buffering synergy is increased by this definition. If both single deletions are already severe, then the buffering synergy obtains a lower weight.

Synergies predicted by FBA and MCT FBA, MoMA, and MCT all predict enzyme synergies, but based on different assumptions and input data. For double inhibitions, all three methods predict, not surprisingly, that cooperating enzymes (i.e., enzymes in one linear pathway) tend to show buffering synergies, while alternative enzymes (e.g., enzymes in alternative pathways) show aggravating synergies. However, these methods explain synergies in different ways. An example is shown in Figure 5 (main text). As expected, a double inhibition that blocks both alternative routes has an aggravating effect, while enzymes within the same linear pathway show buffering synergies. MCT explains this by reference to the control coefficients: in the first case (across alternative pathways), inhibition of one branch increases the flux control of the other branch, while in the second case (within one linear pathway), the first enzyme inhibition decreases the flux control of all other enzymes (see Figure (S4)).

S4.2 Metabolic Fluctuations

Computing the fluctuations caused by chemical noise To model a metabolic pathway under dynamic external perturbations, we may model these perturbations as a random process. The perturbations themselves, and the resulting metabolite fluctuations are described by spectral power density matrices. These matrices resemble the static covariance matrices but are frequency-dependent. If the noise amplitudes are small, we can use a linearised model and compute the spectral densities of concentration fluctuations from the spectral response coefficients [26]

$$\mathcal{S}_{\mathrm{c}}(\omega) = \mathbf{R}^{\mathrm{c}}(\omega)\, \mathcal{S}_{\mathrm{p}}(\omega)\, \mathbf{R}^{\mathrm{c}\dagger}(\omega). \tag{S31}$$

$\mathcal{S}_{\mathrm{c}}(\omega)$ and $\mathcal{S}_{\mathrm{p}}(\omega)$ denote the spectral power densities of metabolites and of perturbation parameters at circular frequency ω, the symbol $\dagger$ indicates the adjoint matrix, and the unscaled first-order spectral response matrices $\mathbf{R}^{\mathrm{c}}(\omega)$ and $\mathbf{R}^{\mathrm{v}}(\omega)$ can be computed from the elasticities [27, 26]. There is a similar formula for fluctuations of the reaction rates.

Chemical noise An important example of random fluctuations is chemical noise. On a microscopic scale, chemical reactions do not run continuously, as assumed in kinetic models, but as discrete random events, converting individual molecules. The resulting random dynamics can be described by a Langevin equation, i.e., a kinetic model with additive noise and separate forward and backward rates [28]. The fluctuations spread in the network, leading to fluctuations of molecule numbers in the macroscopic steady state. In the Langevin equation, the noise term scales with the square root of the mean reaction rate (in units of reaction events per second). Therefore, the smaller the particle numbers, the bigger the relative noise. If the average rates become very small, the approximation breaks down and a more detailed model with discrete reaction events must be used. The fluctuations can be described by Equation (S31), setting $E_{p_{l*}} = \sqrt{\frac{v_{l*}}{N_A \Omega}}$ (where $*$ marks once-way fluxes) and $\mathcal{S}_p(\omega) = \mathbf{I}$, because the chemical fluctuations originate from white noise (see [26]). In practice, the spectral power density of the original noise in reaction l is given by

$$\mathcal{S}_p(\omega) = \frac{v_{+l} + v_{-l}}{N_A\,\Omega} = \frac{\coth(h_l\,\theta_l)\,v_l}{N_A\,\Omega} \approx \frac{1}{N_A\,\Omega}\,\frac{v_l}{h_l\,\theta_l}, \tag{S32}$$

where the approximation holds close to equilibrium (small thermodynamic force θ_l). Mediated by the metabolic dynamics, these input fluctuations lead to fluctuations of metabolite concentrations and fluxes (for an example, see Figure S6). Fast fluctuations are strongly damped: the noise spectrum of the metabolite concentrations decreases at high frequencies, and the system acts as a low-pass filter. Since each reaction rate is also directly affected by its own noise, fluxes also fluctuate at high frequencies. If a stable metabolic state is close to a Hopf bifurcation, it will show a tendency towards oscillations. This becomes apparent in the way the metabolic system transmits random fluctuations, and in its noise spectrum: noise will be amplified around a resonance frequency close to the oscillation frequency after the Hopf bifurcation. All this can be seen from the eigenvalue spectrum of the Jacobian matrix [26].

Limiting behaviour of spectral power densities at high or low frequencies For low or high frequencies, the noise amplitudes can be understood through simple approximations. The spectral power density matrix for metabolite fluctuations has the form $\mathbf{R}^c(\omega)\,\mathbf{M}\,\mathbf{R}^c(\omega)^\dagger$ with a diagonal matrix $\mathbf{M}$. The noise variances for individual metabolites, at specific frequencies, are given by diagonal values

$$\sum_p ||R^c_p(\omega)||^2\, m_p. \tag{S33}$$

The spectral response matrix itself is given by $\mathbf{R}^c_p(\omega) = \mathbf{C}\,(\mathbf{A} - i\,\omega\,\mathbf{I})^{-1}\,\mathbf{B}$, with matrices $\mathbf{C}$ and $\mathbf{B}$ and the Jacobian matrix $\mathbf{A}$. For large frequencies (ω much larger than any eigenvalue of the Jacobian), the term $i\omega\,\mathbf{I}$ dominates and the entire expression (S33) becomes proportional to $\frac{1}{\omega^2}$. High-frequency noise is dominated by direct effects of chemical noise on the adjacent reactant levels, i.e., by fast, non-stationary fluctuations around the stationary fluxes. For low frequencies, in contrast, the spectral power density approaches the variability expected for static variability, and the correlations reflect slow, stationary variations of the stationary fluxes.

Fluctuations on different timescales The amplitude of random fluctuations at specific frequencies are described by the spectral power density. However, in reality, we are usually not interested in the noise level at a precise frequency, but in noise affecting processes on a certain time scale, where much faster noise averages out and much slower noise can be seen as quasi-static. To measure the relevant noise on a time scale of interest, we consider a noisy curve from our model, e.g., the curve of a metabolite concentration, compute a sliding average with a Gaussian kernel (of width τ, e.g., one second), and study how much this average varies across the statistical ensemble at one point in time, or along time in one realisation of the process. Alternatively, we can also consider a sliding average. By changing the width of the kernel, we obtain the variance of our metabolite curve on different time scales. This measure of concentration fluctuations at different time scales can be computed from the spectral power densities using a Fourier transformation (see

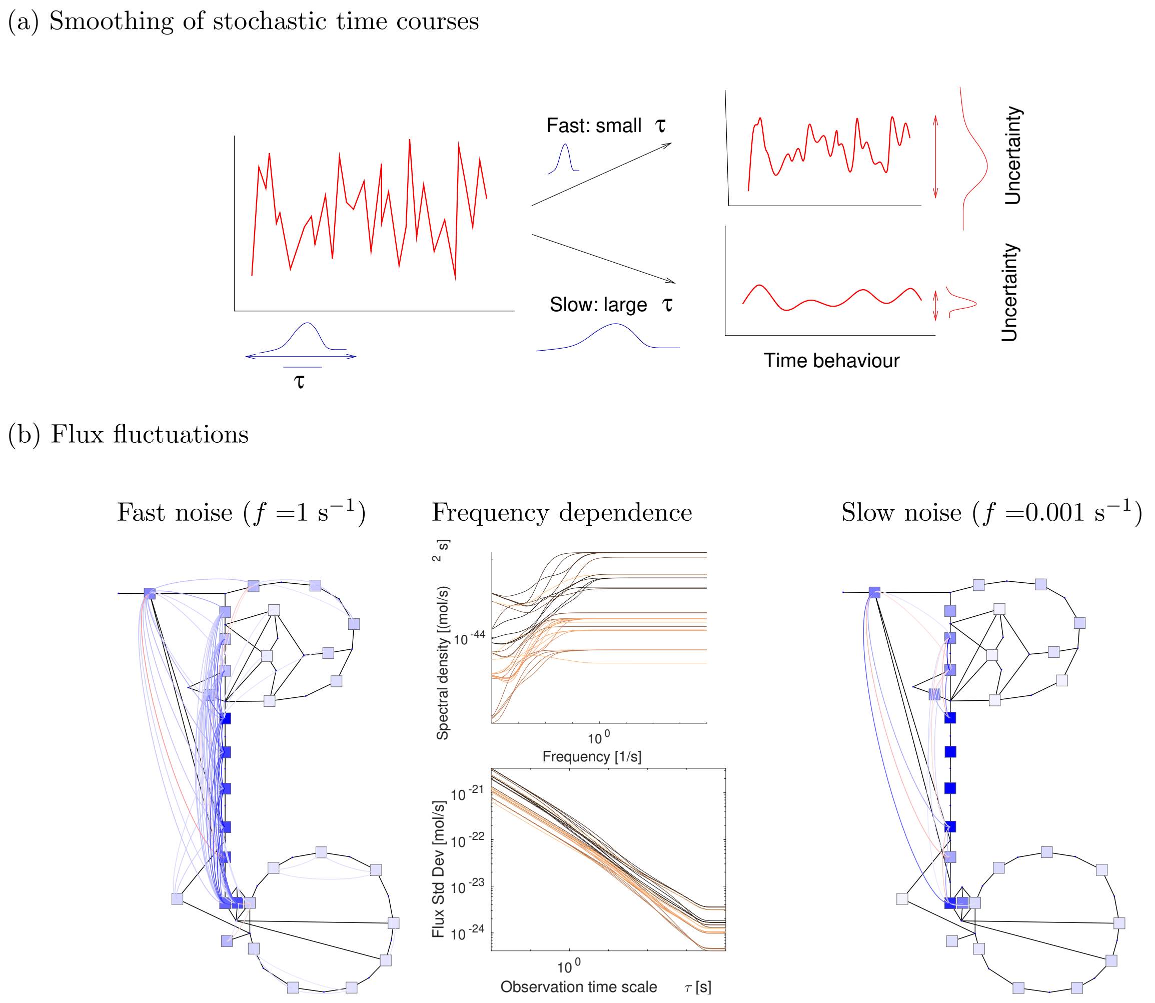

Figure S5: Fluctuations caused by chemical noise. (a) Smoothing of stochastic time courses (e.g., of metabolite concentrations or fluxes) with a Gaussian kernel function leads to "observed" stochastic time courses at a given time resolution τ (reflecting the time resolution of measurements). The variability (red arrows on the right) depends on the time resolution chosen. (b) Flux fluctuations (compare Figure 7 (main text).

Section S5.3).

S4.3 Role of Thermodynamics and Enzyme Saturation in Metabolic Control and Fluctuations

Effect of varying thermodynamic forces Thermodynamic forces and saturation values have effects on metabolic control and fluctuations. Figure S6 shows these effects for a simple model, a pathway of five unimolecular reactions without regulation. In the standard model version, all thermodynamic forces are set to RT and all saturation values are set to $\frac{1}{2}$. To assess the effects of parameter changes, the parameters in the third reaction were systematically varied, setting the force to values of 0.1 RT, 1 RT, and 10 RT, while keeping the metabolite concentrations close to their original values (see Section S3.1). Similarly, the substrate and product saturation values were set to values of 0.1, 0.5, and 0.9. The figure shows static control coefficients and spectral power densities of chemical noise (for metabolite concentrations and fluxes). There are some clear patterns: if the third reaction is close to equilibrium (0.1 RT), it has little control over concentrations and fluxes, and also little influence on the control exerted by other reactions. In contrast, if

(a) Variation of thermodynamic forces

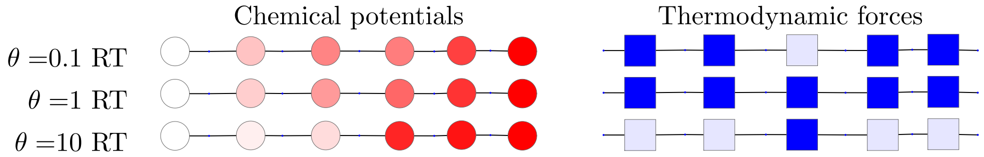

(b) Control coefficients

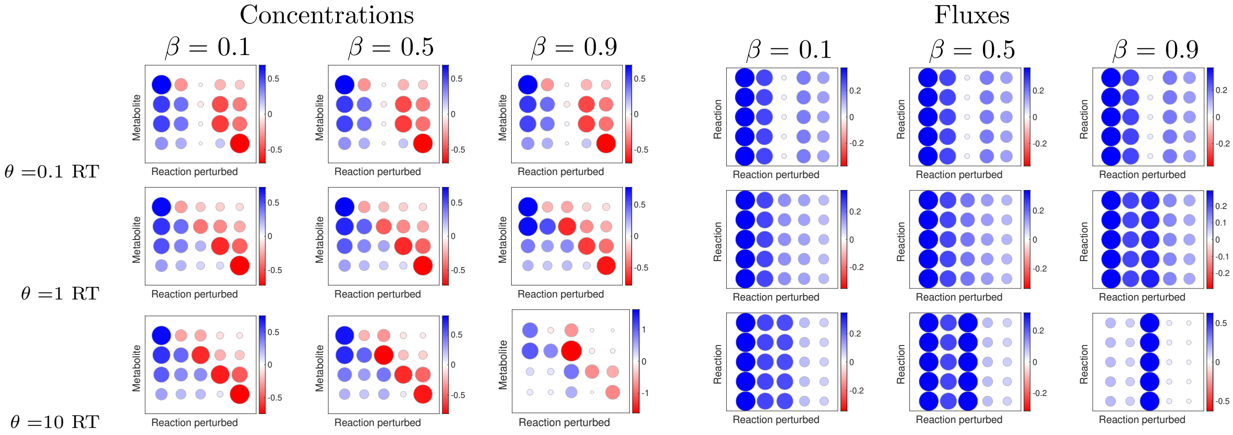

Figure S6: Thermodynamic forces in a linear chain and their effects on metabolic control. Variants of a pathway model with different thermodynamic forces (0.1 RT, RT, 10 RT) and saturation values (0.1, 0.5, 0.9) in the central reaction. (a) The thermodynamic force of the central reaction was made smaller, equal to or larger than in the other reactions by varying the chemical potentials (white: high; red: low). (b) Static control coefficients (for concentrations and fluxes) obtained from different model variants.

the reaction is strongly driven, it exerts a larger control, while the control exerted by downstream enzymes decreases. If the reaction is close to equilibrium, the substrate saturation does not play a role; as the reaction is driven strongly, the substrate saturation increases the control exerted by the reaction. Thus, for a high flux control, reactions must be strongly driven and enzymes must be saturated with substrate.

S5 Mathematical Derivations

S5.1 Probability Densities for Saturation Values and Dissociation Constants

The fraction of enzyme molecules (of one sort of enzyme) bound by a certain metabolite depends on the metabolite concentration c and on the dissociation constant k (indices omitted for simplicity). It can be described by saturation values $\alpha = \frac{1}{1+c/k} = \frac{k}{k+c}$ or $\beta = \frac{c}{k+c} = 1 - \alpha$. The saturation values are directly related to c and k. If α is uniformly distributed in the interval $]0,1[$, the conditional probability densities read

$$p(c) = \frac{k}{(k+c)^2} \quad \text{for fixed}\, k \tag{S34}$$

$$p(k) = \frac{c}{(k+c)^2} \quad \text{for fixed}\, c. \tag{S35}$$

Similar formulae hold for the sampling of β. According to this formula, $\ln(c/k^{\mathrm{M}})$ follows a logistic distribution with location parameter 0 and scale parameter 1. Mean, median, and mode of $\ln(c/k^{\mathrm{M}})$ are given by 0, and the variance is given by $\pi^2/3$

Proof: The probability density of α is $p(\alpha) = 1$. For fixed parameter k, we obtain $\partial\alpha/\partial c = -k/(c+k)^2$ and thus

$$p(c) \quad = \quad C\, p(\alpha) \left|\frac{\partial \alpha}{\partial c}\right| = C \frac{k}{(k+c)^2}. \tag{S36}$$

The normalisation constant C has a value of 1 because

$$1/C = \int_0^\infty \frac{k}{(c+k)^2} \mathrm{d}c = \left.\frac{-k}{k+c}\right|_0^\infty = 1. \tag{S37}$$

For fixed concentration c, we compute $\partial\alpha/\partial k = k/(c+k)^2$ and obtain

$$p(k(\alpha)) \quad = \quad C\, p(\alpha) \left|\frac{\partial \alpha}{\partial k}\right| = C \frac{c}{(k+c)^2}, \tag{S38}$$

again with normalisation constant $C = 1$ because

$$1/C = \int_0^\infty \frac{c}{(k+c)^2} \mathrm{d}k = \left.\frac{k}{c+k}\right|_0^\infty = 1. \tag{S39}$$

S5.2 Metabolic Control and Response Coefficients

Metabolic control and response coefficients (first order) Control and response coefficients describe the effects of small parameter changes on state variables (metabolite concentrations c_i and reaction rates v_l) in a first- or second-order approximation [2, 8]. The unscaled response and control coefficients can be computed from the unscaled elasticities and the stoichiometric matrix [26]. In systems without conservation relations, the first-order unscaled control matrices read

$$\mathbf{C}^{\mathrm{c}} \quad = \quad -(\mathbf{N}\mathbf{E}_{\mathrm{c}})^{-1}\mathbf{N} \tag{S40}$$

$$\mathbf{C}^{\mathrm{v}} \quad = \quad \mathbf{I} + \mathbf{E}_{\mathrm{c}}\, \mathbf{C}^{\mathrm{c}}. \tag{S41}$$

In models with linear conservation relations, the Jacobian $\mathbf{N}\mathbf{E}_{\mathrm{c}}$ is rank-deficient and not invertible, but we can still compute the control coefficients [7]: we select a set of independent internal metabolites (such that the reduced matrix $\mathbf{N}_{\mathrm{R}}$ has full row rank, and the same rank as $\mathbf{N}$). Then, the stoichiometric matrix can be split into a matrix product $\mathbf{N} = \mathbf{L}\,\mathbf{N}_{\mathrm{R}}$, and Equation (S40) is replaced by

$$\mathbf{C}^{\mathrm{c}} = -\mathbf{L}(\mathbf{N}_{\mathrm{R}}\mathbf{E}_{\mathrm{c}}\mathbf{L})^{-1}\mathbf{N}_{\mathrm{R}}. \tag{S42}$$

The response matrices with respect to system parameters p_m read

$$\mathbf{R}^{\mathrm{c}}_{\mathrm{p}} = \frac{\partial \mathbf{s}}{\partial \mathbf{p}} = \mathbf{C}^{\mathrm{c}}\, \mathbf{E}_{\mathrm{p}}, \qquad \mathbf{R}^{\mathrm{v}}_{\mathrm{p}} = \frac{\partial j}{\partial p} = \mathbf{C}^{\mathrm{v}}\, \mathbf{E}_{\mathrm{p}}. \tag{S43}$$

Scaled control and response matrices, e.g., $\hat{R}^{c_i}_{\mathrm{p}} = \partial \ln c_i / \partial \ln p$, are defined similar to the scaled elasticities. Since the enzyme concentrations appear as prefactors in the rate laws, it turns out that scaled response coefficients and scaled control coefficients are identical. With other perturbation parameters (e.g., external metabolite concentrations), this will not be the case.

Metabolic synergy coefficients (second-order response coefficients) For general perturbation parameters u_p and u_q (i.e., enzyme levels or other parameters), we can write the unscaled synergy tensors [10] as

$$R^{c_i}_{u_p u_q} = \sum_k C^{c_i}_{v_l}\,\Gamma^{v_l}_{u_p u_q}, \qquad R^{v_j}_{u_p u_q} = \sum_k C^{v_j}_{v_l}\,\Gamma^{v_l}_{u_p u_q}, \tag{S44}$$

with a tensor Γ defined as

$$\Gamma^{v_l}_{u_p u_q} = \sum_{ij} E^{v_l}_{c_i c_j} R^{c_i}_{u_p} R^{c_j}_{u_q} + \sum_j E^{v_l}_{c_j u_p} R^{c_j}_{u_q} + \sum_i E^{v_l}_{c_i u_q} R^{c_i}_{u_p} + E^{v_l}_{u_p u_q}. \tag{S45}$$

Enzyme levels are parameters with specific properties: there is only one enzyme per reaction, and enzyme levels appear as prefactors in the rate law. Thus, for two enzyme levels e_p and e_q, we obtain the unscaled response coefficients (see Equation (S66))

$$R^{y}_{e_l e_j} = C^{y}_{v_k}\left[E^{v_k}_{c_q c_r}\,C^{c_q}_{v_l}\,C^{c_r}_{v_j}\,v_l\,v_j + \delta_{kj}\,E^{v_k}_{c_q}\,C^{c_q}_{v_l}\,v_l + \delta_{kl}\,E^{v_k}_{c_r}\,C^{c_r}_{v_j}\,v_j\right]\frac{1}{e_l\,e_j}. \tag{S46}$$

The scaled synergy coefficients between a steady-state variable y and enzyme levels e_l and e_j read (see Equation (S67))

$$\begin{aligned}\hat{R}^{y}_{e_l e_j} = & \sum_{kqr}(\hat{C}^{y}_{v_k}\,\hat{E}^{v_k}_{c_q c_r}\,\hat{C}^{c_q}_{v_l}\,\hat{C}^{c_r}_{v_j}) + \sum_q(\hat{C}^{y}_{v_j}\,\hat{E}^{v_j}_{c_q}\,\hat{C}^{c_q}_{v_l}) \\ & + \sum_r(\hat{C}^{y}_{v_l}\,\hat{E}^{v_l}_{c_r}\,\hat{C}^{c_r}_{v_j}) - (\hat{C}^{y}_{v_l}\,\hat{C}^{y}_{v_j}) + (\delta_{lj}\,\hat{C}^{y}_{v_l}).\end{aligned} \tag{S47}$$

The synergy coefficients describe second-order effects of an enzyme (indices $l = j$) or synergies of enzyme pairs (indices $l \neq j$). For enzyme pairs, the Kronecker δ_{lj} vanishes and we can set $\hat{E}^{v_l}_{c_i}\,\hat{C}^{c_i}_{v_j} = \hat{C}^{v_l}_{v_j}$ (see Equation (S40)). By rewriting the second-order elasticities $\hat{E}^{v_k}_{c_q c_r}$ as in Equation (S24) and rearranging Equation (S47), we obtain

$$R^{y}_{e_l e_j} = \hat{C}^{y}_{v_l}\,\hat{C}^{v_l}_{v_j} + \hat{C}^{y}_{v_j}\,\hat{C}^{v_j}_{v_l} - \hat{C}^{y}_{v_l}\,\hat{C}^{y}_{v_j} + \sum_{kqr}\hat{C}^{y}_{v_k}\,\vartheta^{k}_{qr}\,\hat{C}^{v_k}_{v_l}\,\hat{C}^{v_k}_{v_j}. \tag{S48}$$

For mass–action rate laws close to equilibrium and without regulation, the term ϑ^{k}_{qr} is approximately −1.

S5.3 Spectral Power Density and Temporal Variability Due to Chemical Noise

To describe metabolic fluctuations caused by chemical noise, we use the chemical Langevin equation

$$\frac{\mathrm{d}\mathbf{x}}{\mathrm{d}t} = \mathbf{N}\,[\mathbf{a}^{+} - \mathbf{a}^{-}] + \mathbf{N}\left[\mathrm{Dg}\left(\sqrt{\mathbf{a}^{+}}\right)\boldsymbol{\xi}^{+} - \mathrm{Dg}\left(\sqrt{\mathbf{a}^{-}}\right)\boldsymbol{\xi}^{-}\right] \tag{S49}$$

for particle numbers x_i. The reaction propensities $a^{\pm}_l$ denote the probabilities per time (in s^{-1}) for events of reaction l in forward (+) or backward (-) direction, and $\boldsymbol{\xi}^{+}$ and $\boldsymbol{\xi}^{-}$ are vectors of standard Gaussian white noise[5] (in units of $\mathrm{s}^{-1/2}$). In a cell volume Ω, molecule numbers and propensities are related to concentrations and reaction rates as

$$x_i = N_{\mathrm{A}}\,\Omega\,c_i, \qquad a^{\pm}_l = N_{\mathrm{A}}\,v^{\pm}_l, \tag{S50}$$

[5]White noise appears in the formula as the derivative of a Wiener process. It has the covariance function $\mathrm{cov}_{\xi}(\tau) = \langle \xi(t)\,\xi(t+\tau)\rangle_t = \delta(\tau)$ (in s^{-1}) and a spectral power density $S_{\xi}(\omega) = \frac{1}{2\pi}$ (unitless). Note the prefactor convention used for Fourier transforms: $x(t) = \int_{-\infty}^{\infty}\tilde{x}(\omega)\,\mathrm{e}^{i\,\omega\,t}\mathrm{d}\omega$ and $x(\omega) = \frac{1}{2\pi}\int_{-\infty}^{\infty}x(t)\,\mathrm{e}^{-i\,\omega\,t}\mathrm{d}t$.

with Avogadro's constant $N_{\mathrm{A}} \approx 6 \cdot 10^{23}$ mol^{-1}. Using these variables, we can rewrite the chemical Langevin equation as

$$\frac{\mathrm{d}\mathbf{c}}{\mathrm{d}t} = \mathbf{N}\,\frac{1}{\Omega}\,\mathbf{v} + \mathbf{N}\,\frac{1}{\Omega}\,[\mathrm{Dg}\left(\sqrt{\frac{\mathbf{v}_+}{N_{\mathrm{A}}}}\right)\boldsymbol{\xi}^+ - \mathrm{Dg}\left(\sqrt{\frac{\mathbf{v}_-}{N_{\mathrm{A}}}}\right)\boldsymbol{\xi}^-]. \tag{S51}$$

For deviations Δc_i from a stationary state, and setting $\boldsymbol{\xi} = \binom{\boldsymbol{\xi}^+}{\boldsymbol{\xi}^-}$, we can approximate it by

$$\frac{\mathrm{d}\Delta\mathbf{c}}{\mathrm{d}t} = \mathbf{N}\,\mathbf{E}^v_c\,\Delta\mathbf{c} + \mathbf{N}\,\mathbf{E}^v_\xi\,\boldsymbol{\xi}, \tag{S52}$$

with the unscaled elasticity matrices

$$\mathbf{E}^v_c = \frac{1}{\Omega}\,\frac{\partial\mathbf{v}}{\partial\mathbf{c}}, \quad \mathbf{E}^v_\xi = \frac{1}{\Omega\,\sqrt{N_{\mathrm{A}}}}\left(\mathrm{Dg}\left(\sqrt{\mathbf{v}_+}\right), -\mathrm{Dg}\left(\sqrt{\mathbf{v}^-}\right)\right) \tag{S53}$$

in units of s^{-1} and mM s$^{-1/2}$, respectively. Equation (S52) has the form of a standard linear model with perturbation parameters in a vector $\boldsymbol{\xi}$. For this model, we can compute the frequency-response matrices (see [26])

$$\begin{aligned} \mathbf{R}^{\mathrm{c}}_\xi(\omega) &= -\mathbf{L}\,(\mathbf{N}_{\mathrm{R}}\,\mathbf{E}^v_c\mathbf{L} - i\,\omega\mathbf{I})^{-1}\,\mathbf{N}_{\mathrm{R}}\,\mathbf{E}^v_\xi \\ \mathbf{R}^{\mathrm{v}}_\xi(\omega) &= \Omega\left[\mathbf{E}^v_\xi + \mathbf{E}^v_c\,\mathbf{R}^{\mathrm{c}}_\xi(\omega)\right] \end{aligned} \tag{S54}$$

in units of mM s$^{1/2}$ and mol s$^{-1/2}$, respectively. The concentration fluctuations have the spectral power density matrices

$$\mathcal{S}_c(\omega) = \mathbf{R}^{\mathrm{c}}_\xi(\omega)\,\mathcal{S}_c(\omega)\,\mathbf{R}^{\mathrm{c}}_\xi(\omega)^\dagger = \frac{1}{2\,\pi}\,\mathbf{R}^{\mathrm{c}}_\xi(\omega)\,\mathbf{R}^{\mathrm{c}}_\xi(\omega)^\dagger \tag{S55}$$

(in mM^2 s). An analogous formula holds for flux fluctuations (in mol^2 s^{-1}). To study fluctuations on a specific time scale σ (in units of seconds), we consider the fluctuating concentration curve and smoothen it by convolving it with a (normalised) Gaussian function of width σ. The resulting function has the spectral power density

$$\mathcal{S}^{(\sigma)}_c(\omega) = (\mathrm{e}^{-\frac{1}{2}\omega^2\,\sigma^2})^2\,\mathcal{S}_c(\omega). \tag{S56}$$

The function in parenthesis is the Fourier transform of our Gaussian function. The covariance function of the smoothed curve is given by the reverse Fourier transform of the spectral power density

$$\mathrm{cov}^{(\sigma)}_c(\tau) = \int_{-\infty}^{\infty} \mathcal{S}^{(\sigma)}_c(\omega)\,\mathrm{e}^{i\,\omega\,\tau}\,\mathrm{d}\omega. \tag{S57}$$

The variance (the covariance function for time shift $\tau = 0$) is therefore given by

$$\mathrm{cov}^{(\sigma)}_c = \int_{-\infty}^{\infty} \mathcal{S}^{(\sigma)}_c(\omega)\,\mathrm{d}\omega = \frac{1}{2\,\pi}\int_{-\infty}^{\infty} \mathrm{e}^{-\omega^2\,\sigma^2}\,\mathbf{R}^{\mathrm{c}}_\xi(\omega)\,\mathbf{R}^{\mathrm{c}}_\xi(\omega)^\dagger\,\mathrm{d}\omega \tag{S58}$$

An analogous formula holds for the covariance of flux fluctuations.

S5.4 Synergy Effects

Here we derive Equation (S27) for synergy effects. The effect of a double enzyme perturbation on a target variable y can be seen as the sum of three terms: the single-inhibition effects plus a synergy effect η_{ab}:

$$\Delta y^{\mathrm{ab}} = \Delta y_a + \Delta y_b + \eta_{\mathrm{ab}}. \tag{S59}$$

The synergy effect η_{ab}, defined as the difference $\eta_{\mathrm{ab}} = \Delta y^{\mathrm{ab}} - \Delta y_a - \Delta y_b$, can be approximately determined from the metabolic response coefficients. Given a vector $\Delta \mathbf{e}$ of enzyme changes, a second-order Taylor expansion yields

$$y(\mathbf{e} + \Delta \mathbf{e}) \quad \approx \quad y(\mathbf{e}) + \mathbf{R}^y_{\mathbf{e}} \Delta \mathbf{e} + \frac{1}{2} \Delta \mathbf{e}^{\top} \mathbf{R}^y_{uu} \Delta \mathbf{e}. \tag{S60}$$

Therefore, if two enzyme concentrations e_a and e_b are decreased by Δe_a and Δe_b, the target changes by

$$\Delta y^{\mathrm{ab}} \approx -R^y_{e_a} \Delta e_a - R^y_{e_b} \Delta e_b + \frac{1}{2} R^y_{e_a e_a} \Delta e_a^2 + R^y_{e_a e_b} \Delta e_a \Delta e_b + \frac{1}{2} R^y_{e_b e_b} \Delta e_b^2. \tag{S61}$$

The single perturbations yield

$$\begin{aligned} \Delta y_a &\approx -R^y_{e_a} \Delta e_a + \frac{1}{2} R^y_{e_a e_a} \Delta e_a^2 \\ \Delta y_b &\approx -R^y_{e_b} \Delta e_b + \frac{1}{2} R^y_{e_b e_b} \Delta e_b^2. \end{aligned} \tag{S62}$$

With the second-order approximation Equations (S60) and (S61), the synergy effect is given by the unscaled synergy coefficient $R^y_{e_a e_b}$ multiplied by the perturbations:

$$\eta_{\mathrm{ab}} \approx \Delta y^{\mathrm{ab}} - \Delta y_a - \Delta y_b \quad \approx \quad R^y_{e_a e_b} \Delta e_a \Delta e_b. \tag{S63}$$

If all quantities (enzyme levels and target variable) are measured on the logarithmic scale, it is natural to consider the splitting

$$\Delta \log y^{\mathrm{ab}} = \Delta \log y_a + \Delta \log y_b + \eta_{\mathrm{ab}}. \tag{S64}$$

This splitting corresponds to a "null hypothesis" of multiplicative effects and contains the scaled synergy η_{ab}. In the corresponding formulae, the scaled response coefficients are used.

S5.5 Response and Synergy Coefficients

Synergy cofficients for stationary concentrations and fluxes Here, we derive Equation (S47) for scaled synergy coefficients. The unscaled synergy cofficients between general parameters u_l and u_j and state variables y read (as in Equation (S44) and using Einstein's sum convention):

$$R^y_{u_l u_j} = C^y_{v_k} \Gamma^{v_k}_{u_l u_j} = C^y_{v_k} \left[E^{v_k}_{c_q c_r} R^{c_q}_{u_l} R^{c_r}_{u_j} + E^{v_k}_{c_q u_j} R^{c_q}_{u_l} + E^{v_k}_{u_l c_r} R^{c_r}_{u_j} + E^{v_k}_{u_l u_j} \right]. \tag{S65}$$

If the perturbation parameters are enzyme levels e_l and e_j, we can write the elasticities as $E^{v_k}_{c_q e_j} = \delta_{kj}\, \frac{1}{e_j} E^{v_k}_{c_q}$, $E^{v_k}_{c_r e_l} = \delta_{kl}\, \frac{1}{e_l} E^{v_k}_{c_r}$, $E^{v_k}_{e_l e_j} = 0$, and set $R^{c_i}_{e_l} = C^{c_i}_{v_l} \frac{v_l}{e_l}$. By inserting this into Equation (S65), we obtain

$$
\begin{aligned}
R^y_{e_l e_j} &= C^y_{v_k} \left[E^{v_k}_{c_q c_r}\, R^{c_q}_{e_l}\, R^{c_r}_{e_j} + \delta_{kj}\, \frac{1}{e_j} E^{v_k}_{c_q}\, R^{c_q}_{e_l} + \delta_{kl}\, \frac{1}{e_l} E^{v_k}_{c_r}\, R^{c_r}_{e_j} \right] \\
&= C^y_{v_k} \left[E^{v_k}_{c_q c_r}\, C^{c_q}_{v_l}\, C^{c_r}_{v_j}\, v_l\, v_j + \delta_{kj}\, E^{v_k}_{c_q}\, C^{c_q}_{v_l}\, v_l + \delta_{kl}\, E^{v_k}_{c_r}\, C^{c_r}_{v_j}\, v_j \right] \frac{1}{e_l\, e_j}, \qquad \text{(S66)}
\end{aligned}
$$

which is equivalent to the formula given in [10]. The scaled synergy coefficients read, analogous to Equation (S12),

$$
\begin{aligned}
\hat{R}^y_{e_l e_j} &= \frac{e_l\, e_j}{y} R^y_{e_l e_j} - \frac{e_l\, e_j}{y^2} R^y_{e_l} R^y_{e_j} + \delta_{lj} \frac{e_l}{y} R^y_{e_l} \\
&= \frac{1}{y} C^y_{v_k} \left[E^{v_k}_{c_q c_r}\, C^{c_q}_{v_l}\, C^{c_r}_{v_j}\, v_l\, v_j + \delta_{kj} E^{v_k}_{c_q}\, C^{c_q}_{v_l}\, v_l + \delta_{kl} E^{v_k}_{c_r}\, C^{c_r}_{v_j}\, v_j \right] \\
&\quad - \frac{1}{y^2} C^y_{v_l} C^y_{v_j} v_l\, v_j + \delta_{lj} \frac{1}{y} C^y_{v_l} v_l \qquad \text{(S67)}
\end{aligned}
$$

They can be written—again with sum symbols—as

$$
\hat{R}^y_{e_l e_j} = \sum_{krq} (\hat{C}^y_{v_k} \hat{E}^{v_k}_{c_q c_r}\, \hat{C}^{c_q}_{v_l}\, \hat{C}^{c_r}_{v_j}) + \sum_q (\hat{C}^y_{v_j}\, \hat{E}^{v_j}_{c_q}\, \hat{C}^{c_q}_{v_l}) + \sum_r (\hat{C}^y_{v_l}\, \hat{E}^{v_l}_{c_r}\, \hat{C}^{c_r}_{v_j}) - (\hat{C}^y_{v_l} \hat{C}^y_{v_j}) + (\delta_{lj}\, \hat{C}^y_{v_l}). \qquad \text{(S68)}
$$

Response coefficients for general state variables The previous formulae hold for targets y that are stationary concentrations c_i or fluxes v_l. How can we generalise them to other target variables $z(\mathbf{c}, \mathbf{v})$, which are functions of these state variables y_p? Let the unscaled derivatives be called z_{y_p} and $z_{y_p y_q}$. We shall first compute the unscaled, and then the scaled response coefficients of z. The unscaled response coefficients read (with sum convention)

$$
R^z_{e_l} = \frac{\partial z}{\partial e_l} = \frac{\partial z}{\partial y_p} \frac{\partial y_p}{\partial e_l} = z_{y_p}\, R^{y_p}_{e_l}. \qquad \text{(S69)}
$$

We next introduce the scaled derivatives $\hat{z}_{y_p} = \frac{\partial \ln y}{\partial \ln |y_p|}$ and $\hat{z}_{y_p y_q} = \frac{\partial^2 \ln y}{\partial \ln |y_p| \partial \ln |y_q|}$. With their help, we write the unscaled derivatives as

$$
\begin{aligned}
z_{y_p} &= \frac{z}{y_p} \hat{z}_{y_p} \\
z_{y_p y_q} &= \frac{z}{y_p\, y_q} \left[\hat{z}_{y_p y_q} + \hat{z}_{y_p}\, \hat{z}_{y_q} - \delta_{pq} \hat{z}_{y_p} \right]. \qquad \text{(S70)}
\end{aligned}
$$

The unscaled synergy coefficients for our target z read

$$
\begin{aligned}
R^z_{e_l e_j} &= \frac{\partial^2 z}{\partial e_l \partial e_j} = \frac{\partial}{\partial e_j} R^z_{e_l} = \frac{\partial}{\partial e_j} \left[\frac{\partial z}{\partial y_p} R^{y_p}_{e_l} \right] = \left[\frac{\partial}{\partial e_j} \frac{\partial z}{\partial y_p} \right] R^{y_p}_{e_l} + z_{y_p}\, R^{y_p}_{e_l e_j} \\
&= \left[\frac{\partial^2 z}{\partial y_p y_q} \frac{\partial y_q}{\partial e_j} \right] R^{y_p}_{e_l} + z_{y_p}\, R^{y_p}_{e_l e_j} = \left[z_{y_p y_q}\, R^{y_p}_{e_j} \right] R^{y_p}_{e_l} + z_{y_p}\, R^{y_p}_{e_l e_j} \\
&= z_{y_p y_q}\, R^{y_p}_{e_l}\, R^{y_q}_{e_j} + z_{y_p}\, R^{y_p}_{e_l e_j}. \qquad \text{(S71)}
\end{aligned}
$$

Let us now consider the scaled response coefficients. The first-order scaled response coefficients read

$$
\hat{R}^z_{e_l} = \frac{e_l}{z} R^z_{e_l} = \frac{e_l}{z} z_{y_p}\, R^{y_p}_{e_l} = \hat{z}_{y_p}\, \hat{R}^{y_p}_{e_l}. \qquad \text{(S72)}
$$

The scaled synergy coefficients read (compare Equation (S69))

$$\begin{aligned}
\hat{R}^z_{e_l e_j} &= \frac{e_l\, e_j}{z} R^z_{e_l e_j} - \frac{e_l\, e_j}{z^2} R^z_{e_l} R^z_{e_j} + \delta_{lj} \frac{e_l}{z} R^z_{e_l} \\
&= \frac{e_l\, e_j}{z} \left[z_{y_p y_q}\, R^{y_p}_{e_l}\, R^{y_q}_{e_j} + z_{y_p}\, R^{y_p}_{e_l e_j} \right] - R^z_{e_l} R^z_{e_j} + \delta_{lj} R^z_{e_l} \\
&= \left[\hat{z}_{y_p y_q} + \hat{z}_{y_p}\, \hat{z}_{y_q} - \delta_{pq} \hat{z}_{y_p} \right] R^{y_p}_{e_l}\, R^{y_q}_{e_j} + \hat{z}_{y_p}\, R^{y_p}_{e_l e_j} - \hat{z}_{y_p}\, R^{y_p}_{e_l}\, \hat{z}_{y_q}\, R^{y_q}_{e_j} + \delta_{lj} \hat{z}_{y_p}\, R^{y_p}_{e_l} \\
&= \hat{z}_{y_p y_q}\, R^{y_p}_{e_l}\, R^{y_q}_{e_j} + \hat{z}_{y_p}\, R^{y_p}_{e_l e_j} - \delta_{pq} \hat{z}_{y_p}\, R^{y_p}_{e_l}\, R^{y_q}_{e_j} + \delta_{lj} \hat{z}_{y_p}\, R^{y_p}_{e_l}.
\end{aligned} \quad \text{(S73)}$$

For functions $z(\mathbf{c}, \mathbf{v})$ that are multiplicative in fluxes and concentrations (i.e., that are linear if all quantities are given at the logarithmic scale), the first term vanishes.

References

[1] W. Liebermeister, J. Uhlendorf, and E. Klipp. Modular rate laws for enzymatic reactions: thermodynamics, elasticities, and implementation. *Bioinformatics*, 26(12):1528–1534, 2010.

[2] R. Heinrich and S. Schuster. *The Regulation of Cellular Systems.* Chapman & Hall, New York, 1996.

[3] M. Ederer and E.D. Gilles. Thermodynamically feasible kinetic models of reaction networks. *Biophys. J.*, 92:1846–1857, 2007.

[4] R. Wegscheider. Über simultane Gleichgewichte und die Beziehungen zwischen Thermodynamik und Reactionskinetik homogener Systeme. *Z. Phys. Chem.*, 39:257–303, 1902.

[5] S. Schuster and R. Schuster. A generalization of Wegscheider's condition. Implications for properties of steady states and for quasi-steady-state approximation. *J. Math. Chem.*, 3:25–42, 1989.

[6] W. Liebermeister and E. Klipp. Biochemical networks with uncertain parameters. *IEE Proc. Sys. Biol.*, 152(3):97–107, 2005.

[7] C. Reder. Metabolic control theory: a structural approach. *J. Theor. Biol.*, 135:175–201, 1988.

[8] J.-H.S. Hofmeyr. Metabolic control analysis in a nutshell. In *ICSB 2001 Online Proceedings, http://www.icsb2001.org/toc.html*, 2001.

[9] R. Steuer, T. Gross, J. Selbig, and B. Blasius. Structural kinetic modeling of metabolic networks. *PNAS*, 103(32):11868–11873, 2006.

[10] T. Höfer and R. Heinrich. A second-order approach to metabolic control analysis. *J. Theor. Biol.*, 164:85–102, 1993.

[11] J.B.S. Haldane. *Enzymes.* Longmans, Green and Co., London. (republished in 1965 by MIT Press, Cambridge, MA), 1930.

[12] D.A. Beard, E. Babson, E. Curtis, and H. Qian. Thermodynamic constraints for biochemical networks. *J. Theor. Biol.*, 228(3):327–333, 2004.

[13] A. Kümmel, S. Panke, and M. Heinemann. Putative regulatory sites unraveled by network-embedded thermodynamic analysis of metabolome data. *Molecular Systems Biology*, 2:2006.0034, 2006.

[14] A. Hoppe, S. Hoffmann, and H.-G. Holzhütter. Including metabolite concentrations into flux-balance analysis: Thermodynamic realizability as a constraint on flux distributions in metabolic networks. *BMC Syst. Biol*, 1(1):23, 2007.

[15] W. Liebermeister and E. Klipp. Bringing metabolic networks to life: convenience rate law and thermodynamic constraints. *Theor. Biol. Med. Mod.*, 3:41, 2006.

[16] S. Bulik, S. Grimbs, C. Huthmacher, J. Selbig, and H.-G. Holzhütter. Kinetic hybrid models composed of mechanistic and simplified enzymatic rate laws: A promising method for speeding up the kinetic modelling of complex metabolic networks. *FEBS Journal*, 276:410, 2009.

[17] T. Lubitz, M. Schulz, E. Klipp, and W. Liebermeister. Parameter balancing for kinetic models of cell metabolism. *J. Phys. Chem. B*, 114(49):16298–16303, 2010.

[18] Y. Benjamini and Y. Hochberg. Controlling the false discovery rate: a practical and powerful approach to multiple testing. *Journal of the Royal Statistical Society*, Series B 57(1):289–300, 1995.

[19] E.T. Jaynes. Information theory and statistical mechanics. *Physical Review*, 106:620–630, 1957.

[20] W. Liebermeister, E. Klipp, S. Schuster, and R. Heinrich. A theory of optimal differential gene expression. *BioSystems*, 76:261–278, 2004.

[21] C. Gille, C. Bölling, A. Hoppe, S. Bulik, S. Hoffmann, K. Hübner, A. Karlstädt, R. Ganeshan, M. König, K. Rother, M. Weidlich, J. Behre, and H.-G. Holzhütter. HepatoNet1: a comprehensive metabolic reconstruction of the human hepatocyte for the analysis of liver physiology. *Molecular Systems Biology*, 6:411, 2010.

[22] C. Chassagnole, B. Raïs, E. Quentin, D. A. Fell, and J. Mazat. An integrated study of threonine-pathway enzyme kinetics in Escherichia coli. *Biochem J*, 356:415–423, 2001.

[23] R. Chait, A. Craney, and R. Kishony. Antibiotic interactions that select against resistance. *Nature*, 446:668–671, 2007.

[24] D. Segrè, A. DeLuna, G. M. Church, and R. Kishony. Modular epistasis in yeast metabolism. *Nature Genetics*, 37:77–83, 2005.

[25] M. Pellegrini, E.M. Marcotte, M.J. Thompson, D. Eisenberg, and T.O. Yeates. Assigning protein functions by comparative genome analysis: Protein phylogenetic profiles. *PNAS*, 96(8):4285–4288, 1999.

[26] W. Liebermeister. Response to temporal parameter fluctuations in biochemical networks. *J. Theor. Biol.*, 234(3):423–438, 2005.

[27] B.P. Ingalls. A frequency domain approach to sensitivity analysis of biochemical systems. *J. Phys. Chem. B*, 108:1143–1152, 2004.

[28] D.T. Gillespie. The chemical Langevin equation. *J. Chem. Phys.*, 113(1):297–306, 2000.
